# Supplementary material for: A dual-threshold system relying on multiple c-di-GMP metabolic enzymes controls cell fate of a cyanobacterium
Source: PLoS Biol. 2026 Apr 8;24(4):e3003750. doi: 10.1371/journal.pbio.3003750 (PMC13075795; doi:10.1371/journal.pbio.3003750)
Supplement: S1 Fig — (A) A schematic diagram illustrating the primer design strategy for PCR verification. (B)Verification of the deletion of 8 genes in the cdGmax strain via PCR. X: cdGmax, C: WT control. M: DNA marker. The raw images underlying this Figure can be found in S1 Raw Images. (DOCX) [file pbio.3003750.s001.docx]

**
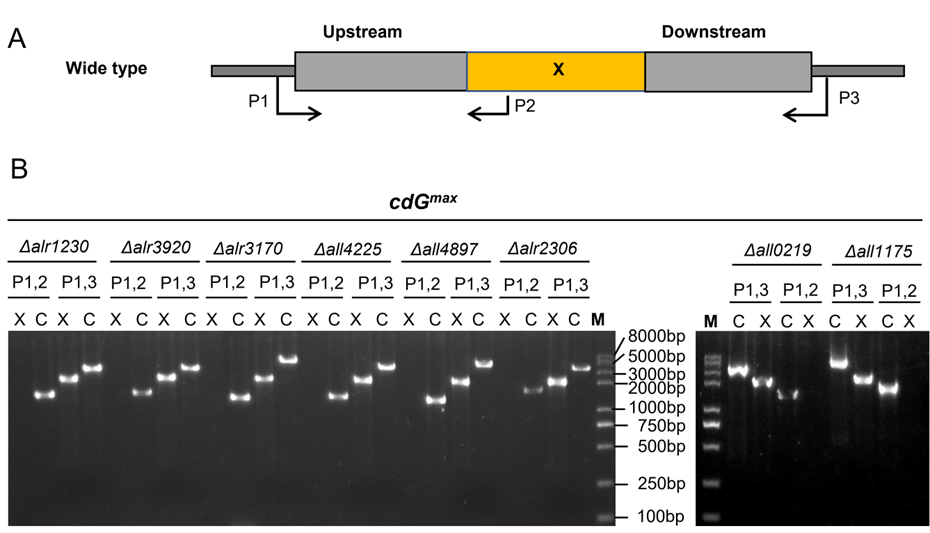
**

**S1 Fig. PCR verification of the *cdG^max^* strain.** (A) A schematic diagram illustrating the primer design strategy for PCR verification. (B)Verification of the deletion of 8 genes in the *cdG^max^* strain via PCR. X: *cdG^max^*, C: WT control. M: DNA marker. The raw images underlying this Figure can be found in S1 Raw images.
