# Supplementary material for: A dual-threshold system relying on multiple c-di-GMP metabolic enzymes controls cell fate of a cyanobacterium
Source: PLoS Biol. 2026 Apr 8;24(4):e3003750. doi: 10.1371/journal.pbio.3003750 (PMC13075795; doi:10.1371/journal.pbio.3003750)
Supplement: S2 Fig — Micrographs of filaments of related PDEs deletion mutant strains. Micrographs of Anabaena filaments of the indicated strain. WT: wild-type. Scale bars: 15 µm. The raw images underlying this Figure can be found in S1 Raw Images. (DOCX) [file pbio.3003750.s002.docx]

**
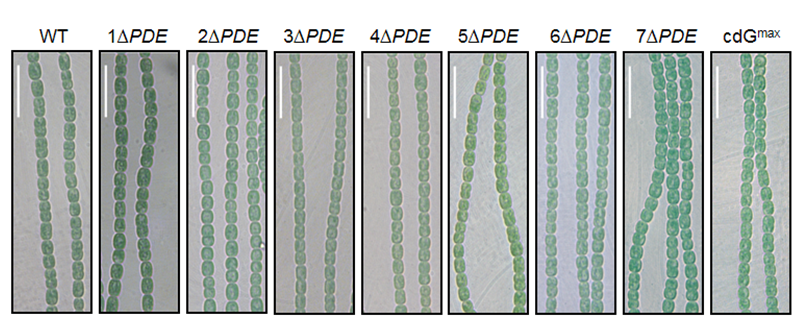
**

**S2 Fig. Micrographs of filaments of related PDEs deletion mutant strains.** Micrographs of *Anabaena* filaments of the indicated strain. WT: wild type. Scale bars: 15 µm. The raw images underlying this Figure can be found in S1 Raw images.
