# Supplementary material for: A dual-threshold system relying on multiple c-di-GMP metabolic enzymes controls cell fate of a cyanobacterium
Source: PLoS Biol. 2026 Apr 8;24(4):e3003750. doi: 10.1371/journal.pbio.3003750 (PMC13075795; doi:10.1371/journal.pbio.3003750)
Supplement: S3 Fig — The initial concentration, measured at OD750 for each strain, was 0.025. The strains were then cultivated in BG11 medium or BG110 medium at the indicated time. All values are shown as mean ± standard deviation, calculated from triplicate data. The data underlying this Figure can be found in S1 Data. (DOCX) [file pbio.3003750.s003.docx]

**
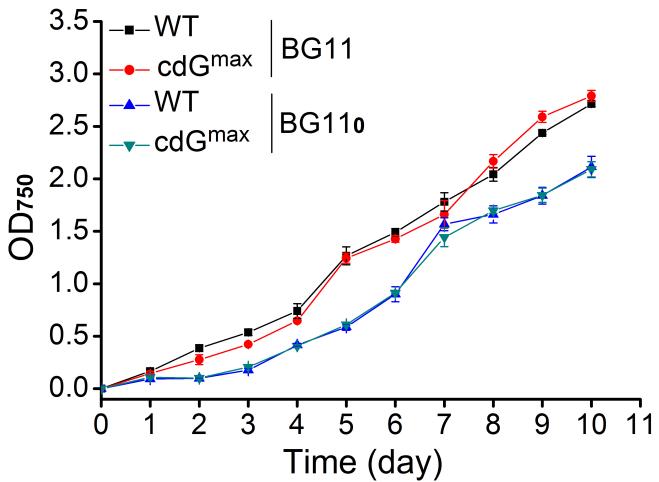
**

**S3 Fig. Growth curves of WT and *cdG^max^*.** The initial concentration, measured at OD750 for each strain, was 0.025. The strains were then cultivated in BG11 medium or BG11_0_ medium at the indicated time. All values are shown as mean ± standard deviation, calculated from triplicate data. The data underlying this Figure can be found in S1 Data.
