# Supplementary material for: A dual-threshold system relying on multiple c-di-GMP metabolic enzymes controls cell fate of a cyanobacterium
Source: PLoS Biol. 2026 Apr 8;24(4):e3003750. doi: 10.1371/journal.pbio.3003750 (PMC13075795; doi:10.1371/journal.pbio.3003750)
Supplement: S4 Fig — (A) A schematic diagram and PCR verification of the conditional control of all1219 in the cdG0 strain. Left panel: the cdG0 strain (14ΔDGC) is a conditional mutant. The promoter region of all1219 was replaced by a Cu2+ and theophylline (TP) inducible platform (CT promoter) at the native chromosomal locus. Right panel, PCR verification of conditional mutant. M: DNA marker. (B) Verification of the deletion of the other 13 genes in the cdG0 strain by PCR. Primers for each gene were designed as depicted in Fig S1A. X: cdG0, C: WT control. M: DNA marker (C) Cultures of the cdG0 strain and WT strain in BG11 medium with or without Cu2+ and TP, which were photographed after 8 days (top) and 20 days (bottom) of incubation. The raw images underlying this Figure can be found in S1 Raw Images. (DOCX) [file pbio.3003750.s004.docx]

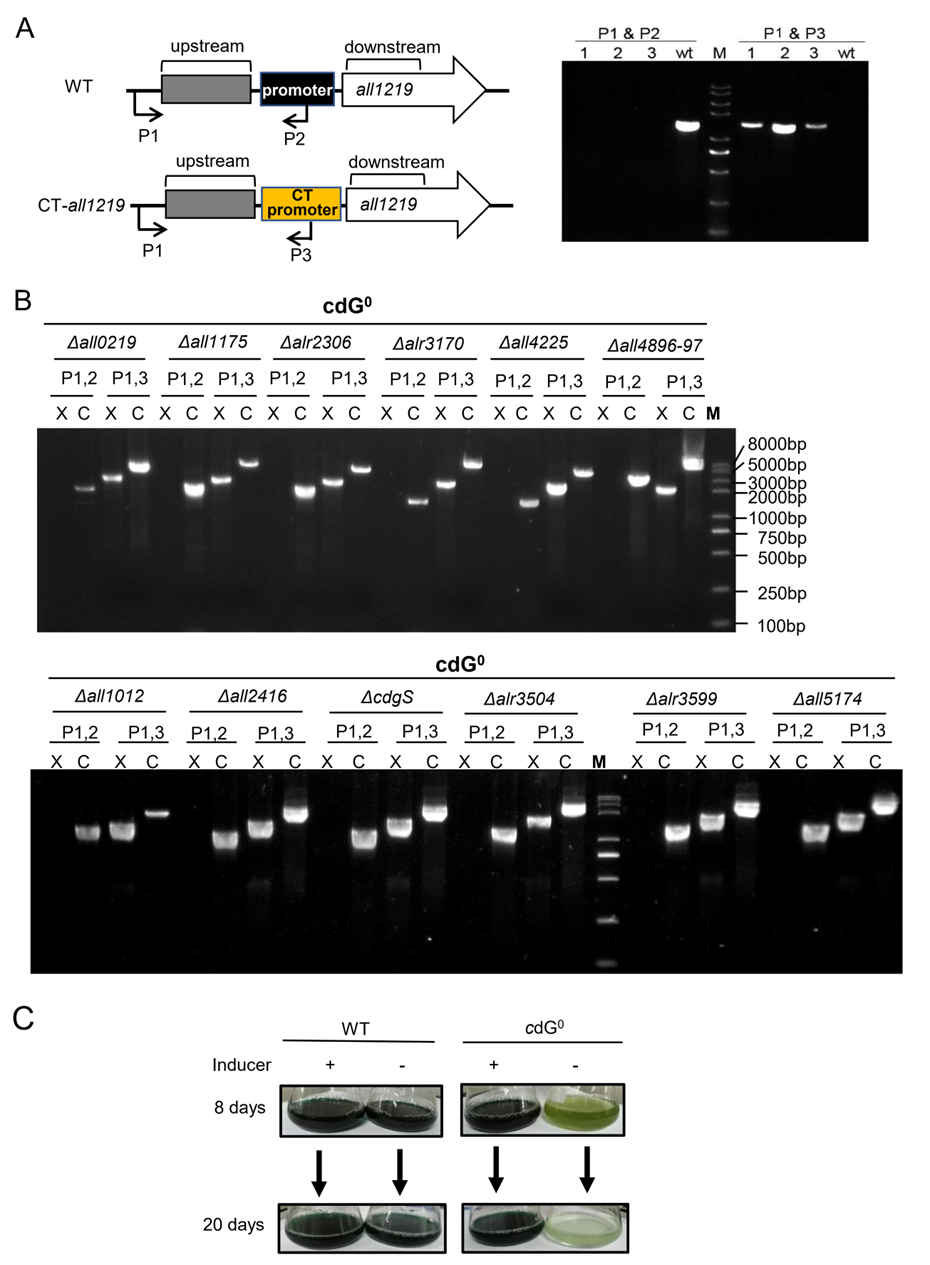


**S4 Fig.** **Verification of a conditional mutant of the *cdG^0^* strain (14*ΔDGC*).** (A) A schematic diagram and PCR verification of the conditional control of *all1219* in *the cdG^0^* strain*.* Left panel: the *cdG^0^* strain (14Δ*DGC*) is a conditional mutant. The promoter region of *all1219* was replaced by a Cu^2+^ and theophylline (TP) inducible platform (CT promoter) at the native chromosomal locus. Right panel, PCR verification of conditional mutant. M: DNA marker. (B) Verification of the deletion of the other 13 genes in the *cdG^0^* strain by PCR. Primers for each gene were designed as depicted in Fig S1A. X: *cdG^0^*, C: WT control. M: DNA marker (C) Cultures of the *cdG^0^* strain and WT strain in BG11 medium with or without Cu^2+^ and TP, which were photographed after 8 days (top) and 20 days (bottom) of incubation. The raw images underlying this Figure can be found in S1 Raw images.
