# Supplementary material for: A dual-threshold system relying on multiple c-di-GMP metabolic enzymes controls cell fate of a cyanobacterium
Source: PLoS Biol. 2026 Apr 8;24(4):e3003750. doi: 10.1371/journal.pbio.3003750 (PMC13075795; doi:10.1371/journal.pbio.3003750)
Supplement: S5 Fig — Micrographs of Anabaena filaments of the indicated strain. Scale bars: 15 µm. WT, wild-type Anabaena. The raw images underlying this Figure can be found in S1 Raw Images. (DOCX) [file pbio.3003750.s005.docx]

**
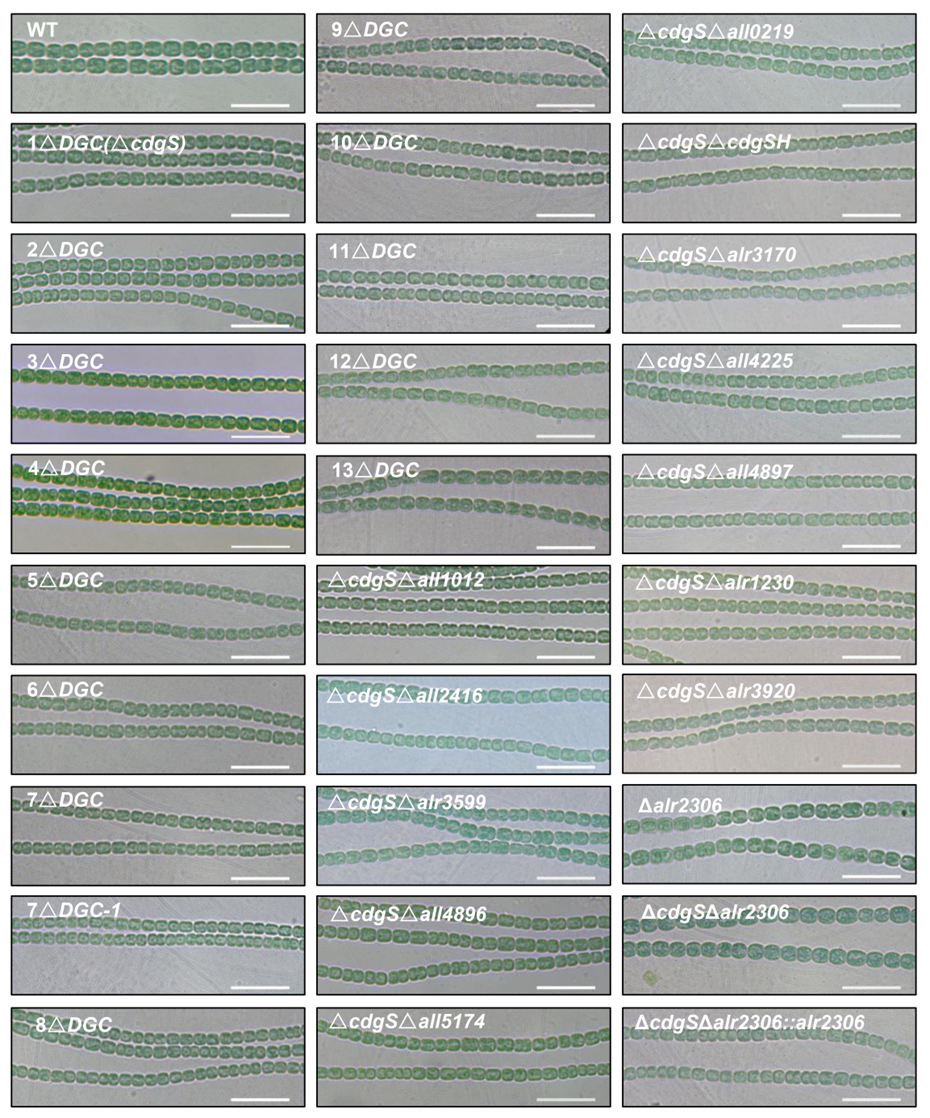
**

**S5 Fig. Micrographs of *Anabaena* filaments of WT and different deletion strains.** Micrographs of *Anabaena* filaments of the indicated strain. Scale bars: 15 µm. WT, wild type *Anabaena*. The raw images underlying this Figure can be found in S1 Raw images.
