# Supplementary material for: A dual-threshold system relying on multiple c-di-GMP metabolic enzymes controls cell fate of a cyanobacterium
Source: PLoS Biol. 2026 Apr 8;24(4):e3003750. doi: 10.1371/journal.pbio.3003750 (PMC13075795; doi:10.1371/journal.pbio.3003750)
Supplement: S6 Fig — (A) The DGC activity of CdgS, All1219-ΔCT, and Alr3599 was evaluated by the reaction product of c-di-GMP. All reaction products are assessed by the retention time of HPLC and correspond to standards. The top panel shows the retention time of the standard nucleotides, including c-di-GMP and GTP. (B) Analysis of the kinetics of c-di-GMP synthesis activity of CdgS, All1219-ΔCT, and Alr3599. The down panel shows curves fitting to the Michaelis–Menten equation by nonlinear regression analysis, and the upper panel shows the enzyme kinetic parameters. Assays were performed using GTP as substrate at varying concentrations. The assays were performed three times, and the average value and standard deviations are shown. (C) Determination of Ki of CdgS, All1219-ΔCT, and Alr3599 for c-di-GMP. Inhibition of the specific activity of CdgS, All1219-ΔCT, and Alr3599 over time was measured in the presence of different c-di-GMP concentrations. Points and error bars represent the mean ± standard deviation (SD) calculated from three biological replicates. The data were fitted to an inhibition model with a variable slope. (D) qRT-PCR analysis of gene alr3599 in the ΔcdgSCT-all1219 strain cultured in BG11 medium with (+) or without (−) Cu2+ and theophylline at the indicated time points. Transcript levels are shown as mean ± SD from three biological replicates. (E) Western blotting analysis of Alr3599 protein levels in the ΔcdgSCT-all1219(alr3599-Flag) strain cultured in BG11 medium with (+) or without (−) Cu2+ and theophylline at the indicated time points. Similar amounts of total proteins extracted from different samples were loaded on the gel, stained with Coomassie Brilliant Blue (Top, CBB), or probed with a polyclonal antibody against Flag (Bottom). M: maker. The data underlying this Figure can be found in S1 Data. The raw images underlying this Figure can be found in S1 Raw Images. (DOCX) [file pbio.3003750.s006.docx]

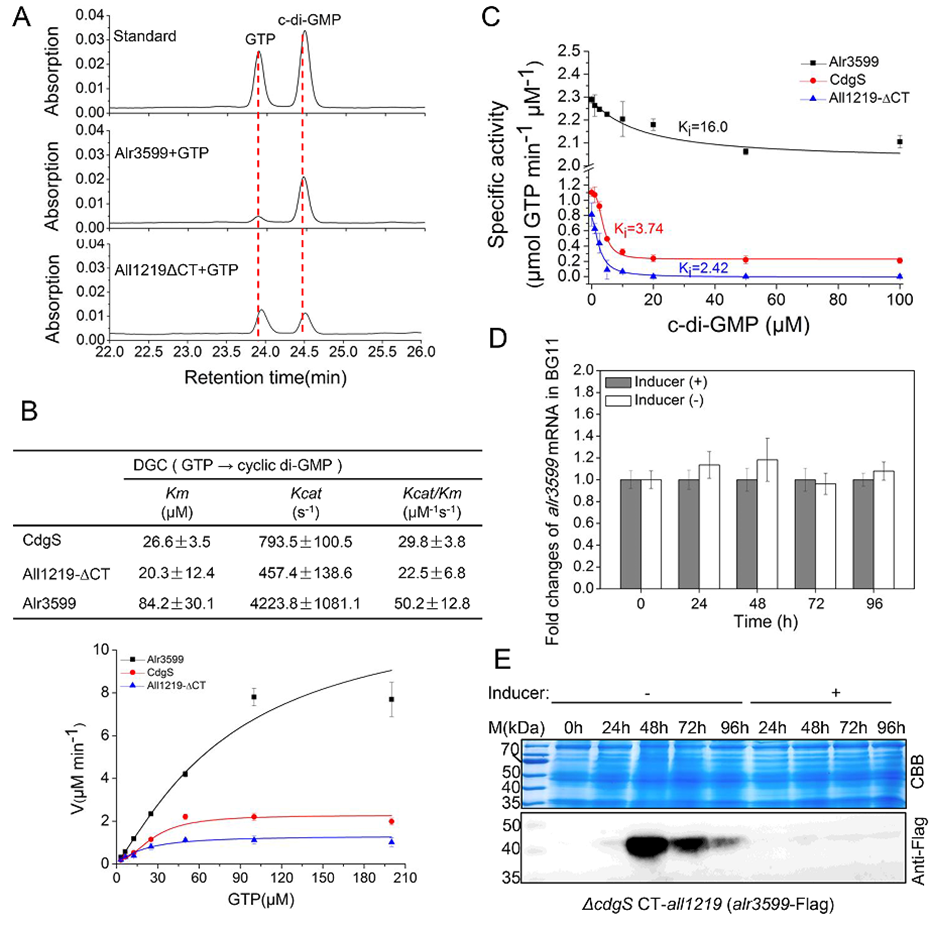


**S6 Fig. The enzymatic characterization of** **CdgS, All1219-****ΔCT, and Alr3599 in vitro.** (A) The DGC activity of CdgS, All1219-ΔCT, and Alr3599 was evaluated by the reaction product of c-di-GMP. All reaction products are assessed by the retention time of HPLC and correspond to standards. The top panel shows the retention time of the standard nucleotides, including c-di-GMP and GTP. (B) Analysis of the kinetics of c-di-GMP synthesis activity of CdgS, All1219-ΔCT, and Alr3599. The down panel shows curves fitting to the Michaelis–Menten equation by non-linear regression analysis, and the upper panel shows the enzyme kinetic parameters. Assays were performed using GTP as substrate at varying concentrations. The assays were performed three times, and the average value and standard deviations are shown. (C) Determination of Ki of CdgS, All1219-ΔCT, and Alr3599 for c-di-GMP. Inhibition of the specific activity of CdgS, All1219-ΔCT, and Alr3599 over time was measured in the presence of different c-di-GMP concentrations. Points and error bars represent the mean ± standard deviation (SD) calculated from three biological replicates. The data were fitted to an inhibition model with a variable slope. (D) qRT-PCR analysis of gene *alr3599* in the *ΔcdgSCT-all1219* strain cultured in BG11 medium with (+) or without (-) Cu^2+^ and theophylline at the indicated time points. Transcript levels are shown as mean ± SD from three biological replicates. (E) Western blotting analysis of Alr3599 protein levels in the *ΔcdgSCT-all1219(alr3599-Flag)* strain cultured in BG11 medium with (+) or without (-) Cu^2+^ and theophylline at the indicated time points. Similar amounts of total proteins extracted from different samples were loaded on the gel, stained with Coomassie Brilliant Blue (Top, CBB), or probed with a polyclonal antibody against Flag (Bottom). M: maker. The data underlying this Figure can be found in S1 Data. The raw images underlying this Figure can be found in S1 Raw images.
