# Supplementary material for: A dual-threshold system relying on multiple c-di-GMP metabolic enzymes controls cell fate of a cyanobacterium
Source: PLoS Biol. 2026 Apr 8;24(4):e3003750. doi: 10.1371/journal.pbio.3003750 (PMC13075795; doi:10.1371/journal.pbio.3003750)
Supplement: S7 Fig — Micrographs of Anabaena filaments of WT and ∆cdgS pICT-all1219 (alr3599-Flag) strains cultured at the indicated times and conditions (with or without inducer (Cu2+ and theophylline (TP)) in BG11 medium). Scale bars represent 15 µm. The raw images underlying this Figure can be found in S1 Raw Images. (DOCX) [file pbio.3003750.s007.docx]

**
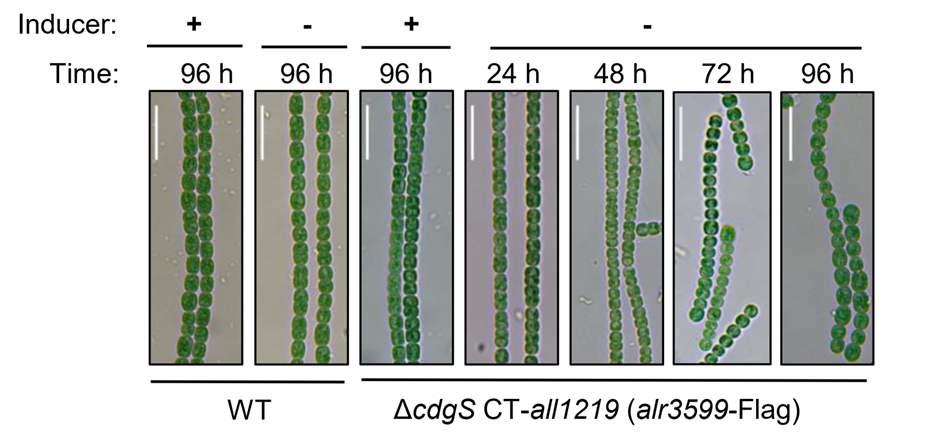
S7 Fig. Micrographs of WT and ∆*cdgS* pICT*-all1219* (*alr3599*-Flag) strains.** Micrographs of *Anabaena* filaments of WT and ∆*cdgS* pICT*-all1219* (*alr3599*-Flag) strains cultured at the indicated times and conditions (with or without inducer (Cu^2+^ and theophylline (TP)) in BG11 medium). Scale bars represent 15µm. The raw images underlying this Figure can be found in S1 Raw images.
