# Supplementary material for: A dual-threshold system relying on multiple c-di-GMP metabolic enzymes controls cell fate of a cyanobacterium
Source: PLoS Biol. 2026 Apr 8;24(4):e3003750. doi: 10.1371/journal.pbio.3003750 (PMC13075795; doi:10.1371/journal.pbio.3003750)
Supplement: S8 Fig — (Upper): representative immunoblot for the quantification of CdgR molecules. Cell lysates derived from a culture normalized to OD₇₅₀ = 0.3 were analyzed alongside a standard curve of purified CdgR (shown on the left) via immunoblotting with anti-CdgR antibodies. Total proteins were loaded and stained with Coomassie Brilliant Blue (CBB). (Lower) The calculated average intracellular concentrations of CdgR and c-di-GMP in WT Anabaena cells, derived from the immunoblot, c-di-GMP quantification, and packed cell volume measurements. The raw images underlying this Figure can be found in S1 Raw Images. (DOCX) [file pbio.3003750.s008.docx]

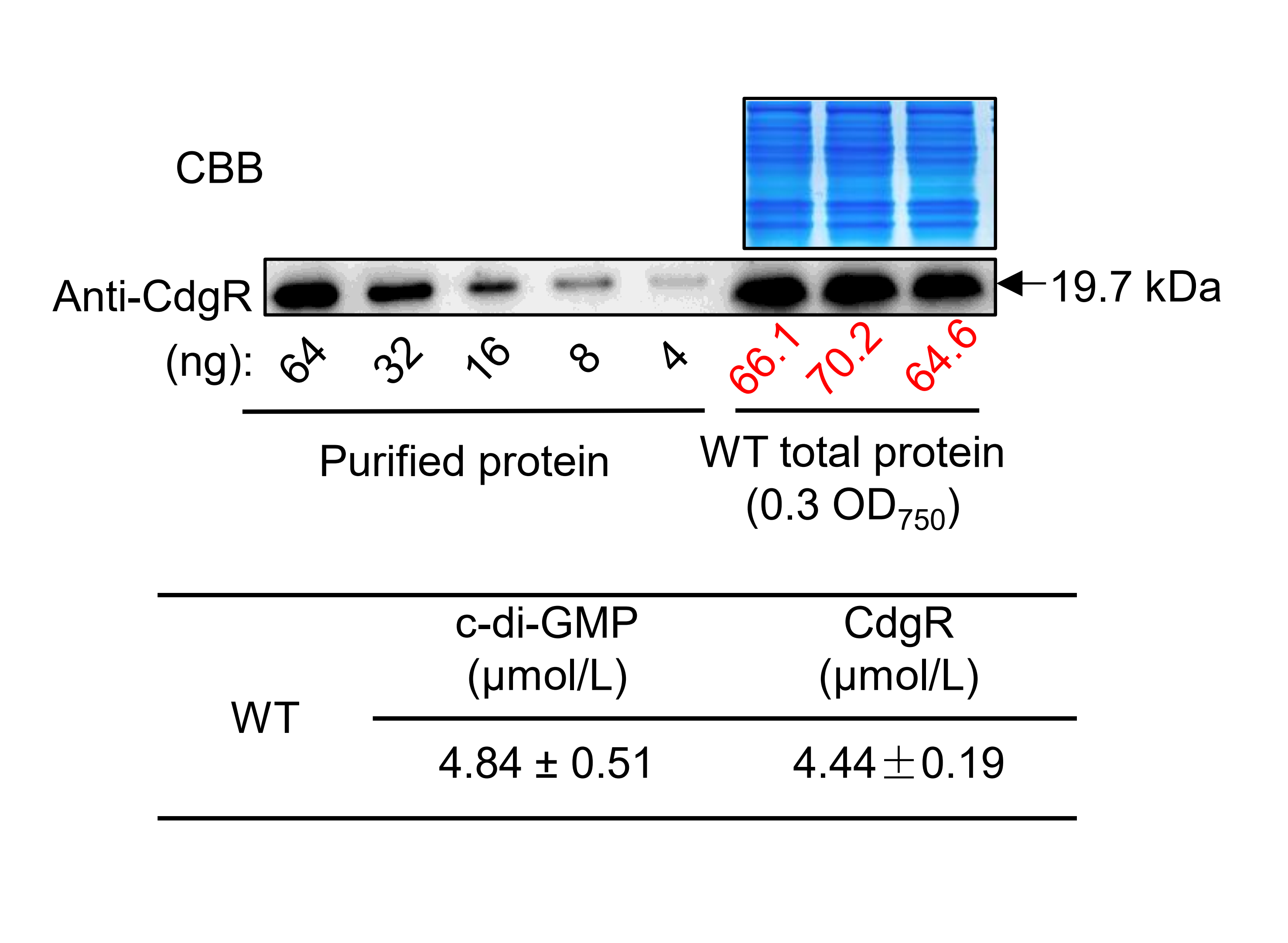


**S8 Fig. Quantification of CdgR and c-di-GMP concentrations in *Anabaena*.**  (Upper): Representative immunoblot for the quantification of CdgR molecules. Cell lysates derived from a culture normalized to OD₇₅₀ = 0.3 were analyzed alongside a standard curve of purified CdgR (shown on the left) via immunoblotting with anti-CdgR antibodies. Total proteins were loaded and stained with Coomassie Brilliant Blue (CBB). (Lower) The calculated average intracellular concentrations of CdgR and c-di-GMP in WT *Anabaena* cells, derived from the immunoblot, c-di-GMP quantification, and packed cell volume measurements. The raw images underlying this Figure can be found in S1 Raw images.
