# Supplementary material for: A dual-threshold system relying on multiple c-di-GMP metabolic enzymes controls cell fate of a cyanobacterium
Source: PLoS Biol. 2026 Apr 8;24(4):e3003750. doi: 10.1371/journal.pbio.3003750 (PMC13075795; doi:10.1371/journal.pbio.3003750)
Supplement: S9 Fig — Bacterial two-hybrid (BACTH) assay for the interaction of the indicated proteins. BTH101 cells producing the indicated proteins fused to either the T18 or the T25 domain of the adenylate cyclase were spotted on plates supplemented with IPTG and X-Gal. The gene encoding the leucine zipper region of GCN4 protein (ZIP) and the empty vectors (EV) were used as positive and negative controls, respectively. Interaction between the two fusion proteins is attested by the blue color of the colony. The experiment was conducted three times, and one representative plate is shown. The raw images underlying this Figure can be found in S1 Raw Images. (DOCX) [file pbio.3003750.s009.docx]

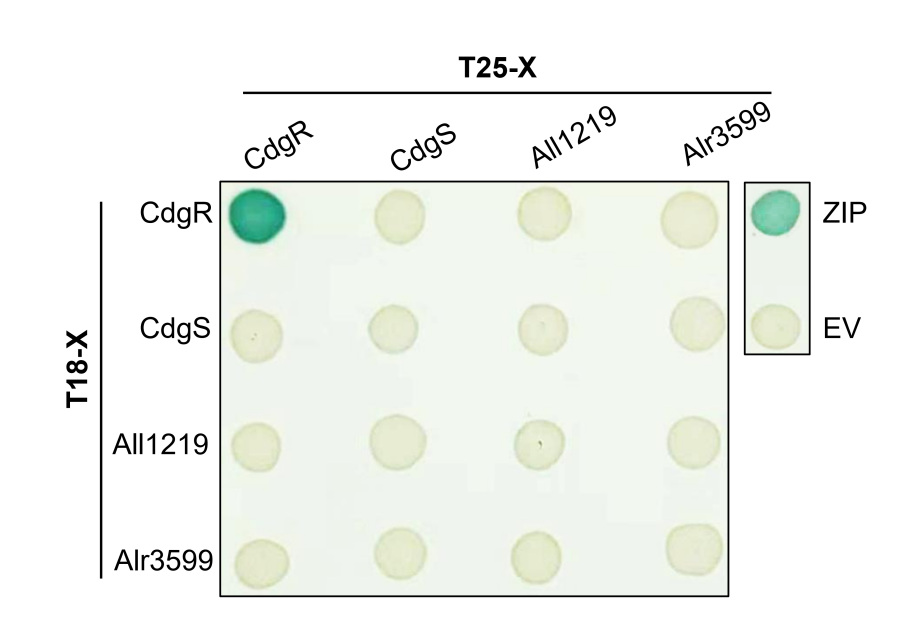


**S9 Fig. Major DGCs are not interacting with the c-di-GMP receptor CdgR.** Bacterial two-hybrid (BACTH) assay for the interaction of the indicated proteins. BTH101 cells producing the indicated proteins fused to either the T18 or the T25 domain of the adenylate cyclase were spotted on plates supplemented with IPTG and X-Gal. The gene encoding the leucine zipper region of GCN4 protein (ZIP) and the empty vectors (EV) were used as positive and negative controls, respectively. Interaction between the two fusion proteins is attested by the blue color of the colony. The experiment was conducted three times, and one representative plate is shown. The raw images underlying this Figure can be found in S1 Raw images.
