# Supplementary material for: A dual-threshold system relying on multiple c-di-GMP metabolic enzymes controls cell fate of a cyanobacterium
Source: PLoS Biol. 2026 Apr 8;24(4):e3003750. doi: 10.1371/journal.pbio.3003750 (PMC13075795; doi:10.1371/journal.pbio.3003750)
Supplement: S10 Fig — (A) Left panel: micrographs of Anabaena filaments of WT and OE- all1549 strains cultured with inducer (1 μM Cu2+ and 2 mM theophylline in BG11 medium). Scale bars represent 15 µm. Right panel: statistical analysis of cell length and cell width of the indicated strains based on images, as shown in the right panel, using a box plot. 200 cells of each strain from three independent experiments were measured. (B) Left panel: micrographs of Anabaena filaments of WT and all1549Ωsp/sm strains cultured in BG11 medium. Scale bars represent 15 µm. Right panel: statistical analysis of cell length and cell width of the indicated strains based on images, as shown in the right panel, using a box plot. 200 cells of each strain from three independent experiments were measured. The data underlying this Figure can be found in S1 Data. The raw images underlying this Figure can be found in S1 Raw Images. (DOCX) [file pbio.3003750.s010.docx]

**
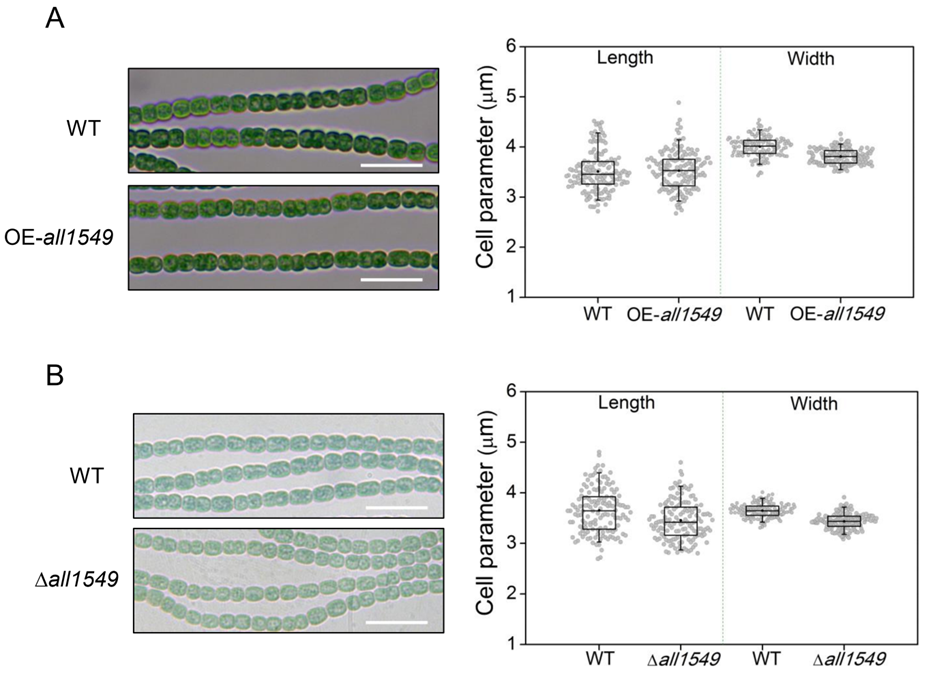
**

**S10 Fig. Overexpression or disruption of All1549 (RelA/SpoT homolog) does not alter cell morphology in *Anabaena*.** (A) Left panel: Micrographs of *Anabaena* filaments of WT and OE- *all1549* strains cultured with inducer (1μM Cu^2+^ and 2 mM theophylline in BG11 medium). Scale bars represent 15µm. Right panel: Statistical analysis of cell length and cell width of the indicated strains based on images, as shown in the right panel, using a box plot. 200 cells of each strain from three independent experiments were measured. (B) Left panel: Micrographs of *Anabaena* filaments of WT and *all1549Ωsp/sm* strains cultured in BG11 medium. Scale bars represent 15µm. Right panel: Statistical analysis of cell length and cell width of the indicated strains based on images, as shown in the right panel, using a box plot. 200 cells of each strain from three independent experiments were measured. The data underlying this Figure can be found in S1 Data. The raw images underlying this Figure can be found in S1 Raw images.
