# Supplementary material for: A dual-threshold system relying on multiple c-di-GMP metabolic enzymes controls cell fate of a cyanobacterium
Source: PLoS Biol. 2026 Apr 8;24(4):e3003750. doi: 10.1371/journal.pbio.3003750 (PMC13075795; doi:10.1371/journal.pbio.3003750)
Supplement: S1 Table — (DOCX) [file pbio.3003750.s011.docx]

**S1 Table . Strains used in this study**

| Strains | Description | Source |
| --- | --- | --- |
| *Escherichia coli* |  |  |
| DH5α | Used for routine transformation |  |
| BL21 (DE3) | Used for protein expression |  |
| Alice | Used for conjugation |  |
| *Anabaena* PCC 7120 | Wild type | Pasteur Culture Collection |
| Δ*all4897* | A markerless deletion mutant by removing the ORF of *all4897* | (24) |
| Δ*all1219* | A markerless deletion mutant by removing the ORF of *all1219* |  |
| Δ*alr3170* | A markerless deletion mutant by removing the ORF of *alr3170* |  |
| Δ*alr2306* (1Δ*PDE*) | A markerless deletion mutant by removing the ORF of *alr2306* |  |
| 2Δ*PDE* | A markerless deletion mutant by removing the ORF of *all0219* in 1Δ*PDE* | This study |
| 3Δ*PDE* | A markerless deletion mutant by removing the ORF of *all4225* in 2Δ*PDE* | This study |
| 4Δ*PDE* | A markerless deletion mutant by removing the ORF of *alr1230* in 3Δ*PDE* | This study |
| 5Δ*PDE* | A markerless deletion mutant by removing the ORF of *alr3920* in 4Δ*PDE* | This study |
| 6Δ*PDE* | A markerless deletion mutant by removing the ORF of *all1175* in 5Δ*PDE* | This study |
| 7Δ*PDE* | A markerless deletion mutant by removing the ORF of *all4897* in 6Δ*PDE* | This study |
| 8Δ*PDE* (cdG^max^) | A markerless deletion mutant by removing the ORF of *alr3170* in 7Δ*PDE* | This study |
| Δ*cdgS* (1Δ*DGC)* | A markerless deletion mutant by removing the ORF of *cdgS* | (24) |
| Δ*cdgS*Δ*alr3504*  (2Δ*DGC*) | A markerless deletion mutant by removing the ORF of *alr3504* in Δ*cdgS* | This study |
| 3Δ*DGC* | A markerless deletion mutant by removing the ORF of *alr3599* in 2Δ*DGC* | This study |
| 4Δ*DGC* | A markerless deletion mutant by removing the ORF of *all2416* in 3Δ*DGC* | This study |
| 5Δ*DGC* | A markerless deletion mutant by removing the ORF of *all1012* in 4Δ*DGC* | This study |
| 6Δ*DGC* | A markerless deletion mutant by removing the ORF of *all5174* in 5Δ*DGC* | This study |
| 7Δ*DGC* | A markerless deletion mutant by removing two genes of *all4896* and *all4897* in 5Δ*DGC* | This study |
| 8Δ*DGC* | A markerless deletion mutant by removing the ORF of *alr3170* in 7Δ*DGC* | This study |
| 8Δ*DGC-1* | A conditional deletion mutant by replacing the promoter of *all1219* with CT promoter in *7*Δ*DGC-1* | This study |
| 9Δ*DGC* | A markerless deletion mutant by removing the ORF of *all0219* in 8Δ*DGC* | This study |
| 10Δ*DGC* | A markerless deletion mutant by removing the ORF of *all4225* in 9Δ*DGC* | This study |
| 11Δ*DGC* | A markerless deletion mutant by removing the ORF of *all1175* in 10Δ*DGC* | This study |
| 12Δ*DGC* | A markerless deletion mutant by removing the ORF of *all5174* in 11Δ*DGC* | This study |
| 13Δ*DGC* | A markerless deletion mutant by removing the ORF of *alr2306* in 12Δ*DGC* | This study |
| 14Δ*DGC* (cdG^0^) | A conditional deletion mutant by replacing the promoter of *all1219* with the CT promoter in 13Δ*DGC* | This study |
| cdG^0^*::ydeH* | The *ydeH* gene, controlled by the Rbcl promoter, is expressed in cdG^0^. | This study |
| cdG^0^*::ydeH^GGAAF^* | Same as cdG^0^*::ydeH*, except that the active site of YdeH was changed from GGDEF to GGAAF. | This study |
| cdG^0^Δ*cdgR* | A markerless deletion mutant by removing the *cdgR* ORF in cdG^0^ | This study |
| Δ*cdgR* | A markerless deletion mutant by removing the *cdgR* ORF in the WT | (7) |
| Δ*cdgS* TC*-all1219* | A conditional deletion mutant by replacing the promoter of *all1219* with the CT promoter in Δ*cdgS* | This study |
| Δ*cdgS* CT*-all1219 (alr3599-Flag)* | A flag tag was inserted into the C-terminal of *alr3599* in Δ*cdgS* CT*-all1219,* at the chromosomal locus | This study |
| Δ*cdgS*Δ*all1012* | A markerless deletion mutant by removing the ORF of *all1012* in Δ*cdgS* | This study |
| Δ*cdgS*Δ*all2416* | A markerless deletion mutant by removing the ORF of *all2416* in Δ*cdgS* | This study |
| Δ*cdgS*Δ*alr3599* | A markerless deletion mutant by removing the ORF of *alr3599* in Δ*cdgS* | This study |
| Δ*cdgS*Δ*all4896* | A markerless deletion mutant by removing the ORF of *all4896* in Δ*cdgS* | This study |
| Δ*cdgS*Δ*all5174* | A markerless deletion mutant by removing the ORF of *all5174* in Δ*cdgS* | This study |
| Δ*cdgS*Δ*all0219* | A markerless deletion mutant by removing the ORF of *all0219* in *ΔcdgS* | This study |
| Δ*cdgS*Δ*cdgSH* | A markerless deletion mutant by removing the ORF of *cdgSH* in Δ*cdgS* | This study |
| Δ*cdgS*Δ*alr2306* | A markerless deletion mutant by removing the ORF of *alr2306* in Δ*cdgS* | This study |
| Δ*cdgS*Δ*alr3170* | A markerless deletion mutant by removing the ORF of *alr3170* in Δ*cdgS* | This study |
| Δ*cdgS*Δ*all4225* | A markerless deletion mutant by removing the ORF of *all4225* in Δ*cdgS* | This study |
| Δ*cdgS*Δ*all4897* | A markerless deletion mutant by removing the ORF of *all4897* in Δ*cdgS* | This study |
| Δ*cdgS*Δ*alr1230* | A markerless deletion mutant by removing the ORF of *alr1230* in Δ*cdgS*. | This study |
| Δ*cdgS*Δ*alr3920* | A markerless deletion mutant by removing the ORF of *alr3920* in ΔcdgS | This study |
| Δ*cdgS*  Δ*alr2306-gfp* | A deletion mutant of *alr2306* in which *alr2306* ORF was replaced by the *gfp* gene in Δ*cdgS* | This study |
| Δ*cdgS*Δ*alr2306*  ::*alr2306* | Δ*cdgS*Δ*alr2306-gfp* mutant complemented by *alr2306* at the native chromosomal locus | This study |
| Δ*all1012*Δ*cdgS* TC*-all1219* | A markerless deletion mutant by removing the ORF of *all1012* in Δ*cdgS*TC-*all1219* | This study |
| Δ*all2416*Δ*cdgS* TC*-all1219* | A markerless deletion mutant by removing the ORF of *all2416* in Δ*cdgS*TC-*all1219* | This study |
| Δ*alr3504*Δ*cdgS* TC*-all1219* | A markerless deletion mutant by removing the ORF of *alr3504* in Δ*cdgS*TC-*all1219* | This study |
| Δ*alr3599*Δ*cdgS* TC*-all1219* | A markerless deletion mutant by removing the ORF of *alr3599* in Δ*cdgS*TC-*all1219* | This study |
| Δ*all4896*Δ*cdgS* TC*-all1219* | A markerless deletion mutant by removing the ORF of *all4896* in Δ*cdgS*TC-*all1219* | This study |
| Δ*all5174*Δ*cdgS* TC*-all1219* | A markerless deletion mutant by removing the ORF of *all5174* in Δ*cdgS*TC-*all1219* | This study |
| OE-*all1549* | The *all1549* gene, controlled by the CT promoter, expressed in WT. | (49) |
| *all1549Ωsp/sm* | Disruption *rel_ana_*; Sp^r^ Sm^r^ |  |
