# Supplementary material for: A dual-threshold system relying on multiple c-di-GMP metabolic enzymes controls cell fate of a cyanobacterium
Source: PLoS Biol. 2026 Apr 8;24(4):e3003750. doi: 10.1371/journal.pbio.3003750 (PMC13075795; doi:10.1371/journal.pbio.3003750)
Supplement: S2 Table — (DOCX) [file pbio.3003750.s012.docx]

**S2 Table. Primers used in this study (sequence in minuscule corresponds to the overlapping homologous parts in PCR fragments for ligation during cloning, and the sequence in capital letters corresponds to those used for DNA amplification by PCR).**

| **Name** | **Sequence (5’-3’)** |  |
| --- | --- | --- |
| P*all1219*F1572m | gcagaaattcgatatctagatctATCCCCAATCCTTAATACCCAATC | pCpf1b-ICT-  *all1219*-R126m |
| P*all1219*R550m | ggctcgactctagctagagGTAGAAGTTACCTAAAGTACTTGAAGTTG |  |
| PcoquwF | ctctagctagagtcgagccCGTTAAGGGATTTTGGTCATGAG |  |
| PV_19 | CATCTTGTTGTTACCTCCTTAGCA |  |
| P*all1219*F1 | tgctaaggaggtaacaacaagATGAATAAGCAACTAGGGAATCCTCT |  |
| P*all1219*R836 | aacgttgttgccattgcggatccTCTATCAAACTGCGCGGAACT |  |
| cr_ *all1219*R126mF | agatTTTCTTCATGATTGTGAATGAT |  |
| cr_ *all1219*R126mR | agacATCATTCACAATCATGAAGAAA |  |
| P*ydeh*-F1 | cttggaaggatactcgagATGATCAAGAAGACAACGGAAATTG | pRbcl-*ydeH* |
| P*ydeh*-R1 | aacaaaacgcttgggcttCTAAACTCGGTTAATCACATTTTGTTC |  |
| P25TNotI-F | AAGCCCAAGCGTTTTGTTATTGG |  |
| PPrbcl-R | CTCGAGTATCCTTCCAAGATGTC |  |
| P*ydeh*-F2 | atcgctacGGGGGCGCAGCATTTATCATTATTGTCAAAG | pRbcl-  *ydeH*^GGAAF^ |
| P*ydeh*-R2 | caataatgatAAATGCTGCGCCCCCGTAGCGATAAACCGTTTCGTA |  |
| P*alr2306*F893m | gcagaaattcgatatctagatctTCATTAGTGTGGGCGAAGAA | pCpf1b-  M*alr2306-gfp-*  *F580* |
| P*alr2306*R1m-gfp | GAGGCCTTGGATCCAGTCATTCTATATTTGTTTTAGTTAGTGCCATAG |  |
| P*alr2306*F1833-gfp | gtggtagcactagcgtcggtTGGTAGTTTAGGTTGATACTCCTGA |  |
| P*alr2306*R2661 | aacgttgttgccattgcggatccATGTTGGATTGGGATACTCCTGT |  |
| PYFP2-seF | ATGACTGGATCCAAGGCCTCT |  |
| PYFP2-seR | ACCGACGCTAGTGCTACC |  |
| cr_ *alr2306*F580F | agatTCAGGAGATTAGGAAAAGCTTT |  |
| cr_ *alr2306*F580R | agacAAAGCTTTTCCTAATCTCCTGA |  |
| P*alr2306*F893m | gcagaaattcgatatctagatctTCATTAGTGTGGGCGAAGAA | pCpf1b-  M*alr2306*-gfpF304 |
| P*alr2306*R2661 | aacgttgttgccattgcggatccATGTTGGATTGGGATACTCCTGT |  |
| cr-gfpF304F | agatTCCTGATCACATGAAACGGCAT |  |
| cr-gfpF304R | agacATGCCGTTTCATGTGATCAGGA |  |
| P*alr3599*F567 | gcagaaattcgatatctagatctTACAGCAAGCACAAGTGTATAAACA | pCpf1b-  M*alr3599c-flag*-F9C |
| P*alr3599*-FLAGR1 | ATCACCGTCATGGTCTTTGTAGTCTTGACTATCTTCCCATTGACA |  |
| P*alr3599*-FLAGR2 | TCATCCTTGTAGTCGATGTCATGATCTTTATAATCACCGTCATGGTCTTTGTAGTC |  |
| P*alr3599*-FLAGR3 | AGAGATTGGGGATTGGCTACTTGTCATCGTCATCCTTGTAGTCGATGTCA |  |
| P*alr3599*F1073 | CCAATCCCCAATCTCTAAGATGGATAA |  |
| P*alr3599*R1623 | aacgttgttgccattgcggatccACAAGCCCGCACTCAAGC |  |
| cr_ *alr3599*F9CF | agatTTTTCCCCTTGCGCCCTACCAG |  |
| cr_ *alr3599*F9CR | agacCTGGTAGGGCGCAAGGGGAAAA |  |
| PV_3 | CATGGTATATCTCCTTCTTAAAGTT | pHTS-  All1219-∆CT |
| PV_6 | GGTTCTGGTGGTGGTAGCA |  |
| P*all1219*-F1243 | aggagatataccatgATGCAGGAAGAGTTAAAGCG |  |
| P*all1219*-R2214 | accaccaccagaaccGAACTCAGTATTTACCCTATC |  |
| p*alr3599*F1 | aggagatataccatgATGTCAGGCATAAGCCCATAT | pHTS-Alr3599 |
| p*alr3599*R1029 | accaccaccagaaccACTATCTTCCCATTGACAAATACG |  |
| P*allrs04*F334 | CCAGTTCCGCTATCAGAGAG | qRT-PCR |
| P*allrs04*R456 | GAGGAGAGAGTTGGTGGTAAG |  |
| Q-*alr3599*F | CGGTGATATCTAGATTGTGGGAAC |  |
| Q-*alr3599*R | GCAGCTTCTAACTGTTTATACACTTG |  |
| P*all1219*hyF | atcacctctagtggtgaaATGAATAAGCAACTAGGGAATCCT | pUT18C / pKT25-*all1219* |
| P*all1219*hyR | gatgtcgatctagatctcTCAGAACTCAGTATTTACCCTATCCG |  |
| P*alr3599*hyF | atcacctctagtggtgaaATGTCAGGCATAAGCCCATATTC | pUT18C / pKT25-*alr3599* |
| P*alr3599*hyR | gatgtcgatctagatctcCTATTGACTATCTTCCCATTGACAAATA |  |
| P*cdgS*hyF | atcacctctagtggtgaaATGAAAAATACAGTTCCAGAGAGC | pUT18C / pKT25-*cdgS* |
| P*cdgS*hyR | gatgtcgatctagatctcTTAAGCTAGTCGATGGTGAGC |  |
| all1219-P1 | CCTTATTCGCCATGCTACTACTG | CT-all1219 |
| all1219-P2 | ACGCAGGCTTAAGCCTAAC |  |
| all1219-P3 | TAGGGAATTCGGTGATACCAG |  |
| all1012-P1 | ACTATGCGGTGATGTCCAGAG | Δall1012 |
| all1012-P2 | CAATTACCCCACAGCCCATAC |  |
| all1012-P3 | GGAAGAAGAAGTCTCGCAAAACC |  |
| all2416-P1 | TCCCATTCCCCAGTTCCCA | Δall2416 |
| all2416-P2 | ACCTTGGTGAAGTGCTGT |  |
| all2416-P3 | CTGGTGTAGCGATATCAACGATA |  |
| alr3504-P1 | AGTTGTTTCCTTGAGGCTTGATG | Δalr3504 |
| alr3504-P2 | GATTGGTCAACCCAGTGAGA |  |
| alr3504-P3 | AACCCCGTACTGTCCAGATG |  |
| alr3599-P1 | GTATCGATCAAGTGTTGGGCTT | Δalr3599 |
| alr3599-P2 | ACGATCAACAGATTCTGCATCAT |  |
| alr3599-P3 | CCATCGACACCTGTCAGTCTAT |  |
| all4225-P1 | TCCACCTGTCGCCCTGTCA | Δall4225 |
| all4225-P2 | AGTCTCGCAACGTGGCGGATGT |  |
| all4225-P3 | TCCTACAGGCCCCGCAGAT |  |
| all5174-P1 | AACAATAATGGGCTGTGGGAATA | Δall5174 |
| all5174-P2 | CCAGCTTAGGCAAGATCAAATC |  |
| all5174-P3 | ATTGGGATGGCGAAAACGAT |  |
| all4896-P1 | TGGAGAACCATGAATCAGCCA | Δall4896 |
| all4896-P2 | CAAATCCGCGATCACTCGT |  |
| all4896-P3 | GGATGTAAATTGACCCGTGACA |  |
| all4897-P1 | TCACTGCCCCGTGGTACAT | Δall4897 |
| all4897-P2 | TGCGGGTTTACCCGTGAGGAGA |  |
| all4897-P3 | TACCAGCCGCCCTTGTTCTG |  |
| all0219-P1 | GGTAAGACAGATCGCTCCGT | Δall0219 |
| all0219-P2 | AGTACATCATGTAGGGCATCAT |  |
| all0219-P3 | CTGCACCCAAAGTGGAAGGT |  |
| alr1230-P1 | TCAGCCAACAGCGACTACA | Δalr1230 |
| alr1230-P2 | CCGTGAGGAAAATGAAAGGAAT |  |
| alr1230-P3 | TGTGCTGCTGTAATTCCTCG |  |
| alr2306-P1 | CTGCCAGCGTTGTGTAGTTC | Δalr2306 |
| alr2306-P2 | AAAGCTTTTCCTAATCTCCTGA |  |
| alr2306-P3 | AGGCGCTGTCTATTCCCTGAT |  |
| alr3170-P1 | AGGTCGCTGGCGAATACTG | Δalr3170 |
| alr3170-P2 | AATATGGAGTTTTCATAAGACT |  |
| alr3170-P3 | ACCCAGGGTATACAGGGAACA |  |
| alr3920-P1 | TGTCCGCAATTATGGAGTGTG | Δalr3920 |
| alr3920-P2 | TAGCACTGTGATCATCGACTAC |  |
| alr3920-P3 | GACTGTGACATTTGCACCCA |  |
| all1175-P1 | GTAGCCAGTCTGATGACACT | Δall1175 |
| all1175-P2 | ACTGTACCAACGTGATAATTC |  |
| all1175-P3 | CAGAGGGTTAACCGGACTTG |  |
| cdgS-P1 | TGGACGGTCTGACACTGGCA | ΔcdgS |
| cdgS-P2 | ACGATATCAGGTTGCAGTTCTT |  |
| cdgS-P3 | GTTGGGCAGAAAGAAGCGGA |  |
