# Supplementary material for: A dual-threshold system relying on multiple c-di-GMP metabolic enzymes controls cell fate of a cyanobacterium
Source: PLoS Biol. 2026 Apr 8;24(4):e3003750. doi: 10.1371/journal.pbio.3003750 (PMC13075795; doi:10.1371/journal.pbio.3003750)
Supplement: S3 Table — (DOCX) [file pbio.3003750.s013.docx]

**S3 Table . Plasmids used in this study**

| Plasmids | Description | Source |
| --- | --- | --- |
| CRISPR-pCpf1b | Sp^r^ Sm^r^; vector carrying the CRISPR-pCpf1b genome editing system | (29) |
| pCpf1b-*McdgS*-F149 | Sp^r^ Sm^r^; for constructing mutant Δ*cdgS* by CRISPR-pCpf1b genome editing system, originated from CRISPR-pCpf1b | (26) |
| pHTS-*cdgS* | Km^r^ ; plasmid for the expression of the C-terminal Strep-tagged CdgS |  |
| pCpf1b-ICT-*all1219*-R126m | Sp^r^ Sm^r^; for constructing a conditional mutant strain of CT-*all1219* by CRISPR-pCpf1b genome editing system, originated from CRISPR-pCpf1b | This study |
| pRbcl-*ydeH* | Kmr Nmr; plasmid for overexpression of ydeH using *rbcl* promoter | This study |
| pRbcl-*ydeH^GGAAF^* | Kmr Nmr; plasmid for overexpression of ydeH encoding a product with an inactivated c-di-GMP synthetase domain | This study |
| pCpf1b-M*alr2306*-gfp-F580 | Sp^r^ Sm^r^; for constructing Δ*alr2306-gfp* by CRISPR-pCpf1b genome editing system, based on CRISPR-pCpf1b | This study |
| pCpf1b-M*alr2306*-gfpF304 | Sp^r^ Sm^r^; for constructing a complementary strain of *alr2306* by CRISPR-pCpf1b genome editing system, based on CRISPR-pCpf1b | This study |
| pCpf1b-M*alr3599c*-*flag*-F9C | Sp^r^ Sm^r^; for constructing *alr3599c-flag* by CRISPR-pCpf1b genome editing system, based on CRISPR-pCpf1b | This study |
| pHTS-All1219-∆CT | Km^r^ ; plasmid for the expression of the C-terminal Strep-tagged All1219 without CHASE2 and TMs domain. | This study |
| pHTS-Alr3599 | Km^r^ ; plasmid for the expression of the C-terminal Strep-tagged Alr3599*.* | This study |
| pCpf1b-M*all1219*-F172 | Sp^r^ Sm^r^; for constructing Δ*all1219* by CRISPR-pCpf1b genome editing system, origin from CRISPR-pCpf1b | (24) |
| pCpf1b-M*alr3599*-F489 | Sp^r^ Sm^r^; for constructing Δ*alr3599* by CRISPR-pCpf1b genome editing system, origin from CRISPR-pCpf1b |  |
| pCpf1b-cdgR–R419 | Sp^r^ Sm^r^; for constructing Δ*cdgR* by CRISPR-pCpf1b genome editing system, based on CRISPR-pCpf1b | (7) |
| PCT | Km^r^ Nm^r^; expression plasmid with the CT promoter for *Anabaena* | (52) |
| pUT18C | Amp^r^; Two-hybrid plasmid, N-terminal cyaA (T18) fusion |  |
| pKT25 | Km^r^; Two-hybrid plasmid, C-terminal cyaA (T25) fusion |  |
| pUT18C-*zip* | A derivative of pUT18C in which the leucine zipper of GCN4 is genetically fused in-frame to the T18 fragment |  |
| pKT25-*zip* | A derivative of pKT25 in which the leucine zipper of GCN4 is genetically fused in frame to the T25 fragment |  |
| pUT18C-*cdgR* | Amp^r^; pUT18C carrying *cdgR* (T18 at the N-terminal) | (7) |
| pKT25-*cdgR* | Km^r^; pKT25 carrying *cdgR* (T25 at the N-terminal) |  |
| pUT18C-*cdgS* | Amp^r^; pUT18C carrying *cdgS* (T18 at the N-terminal) | This study |
| pUT18C-*all1219* | Amp^r^; pUT18C carrying *all1219* (T18 at the N-terminal) | This study |
| pUT18C-*alr3599* | Amp^r^; pUT18C carrying *alr3599* (T18 at the N-terminal) | This study |
| pKT25-*cdgS* | Km^r^; pKT25 carrying *cdgS* (T25 at the N-terminal) | This study |
| pKT25-*all1219* | Km^r^; pKT25 carrying *all1219* (T25 at the N-terminal) | This study |
| pKT25-*alr3599* | Km^r^; pKT25 carrying *alr3599* (T25 at the N-terminal) | This study |
