# Supplementary material for: A dual-threshold system relying on multiple c-di-GMP metabolic enzymes controls cell fate of a cyanobacterium
Source: PLoS Biol. 2026 Apr 8;24(4):e3003750. doi: 10.1371/journal.pbio.3003750 (PMC13075795; doi:10.1371/journal.pbio.3003750)

Fig. 2

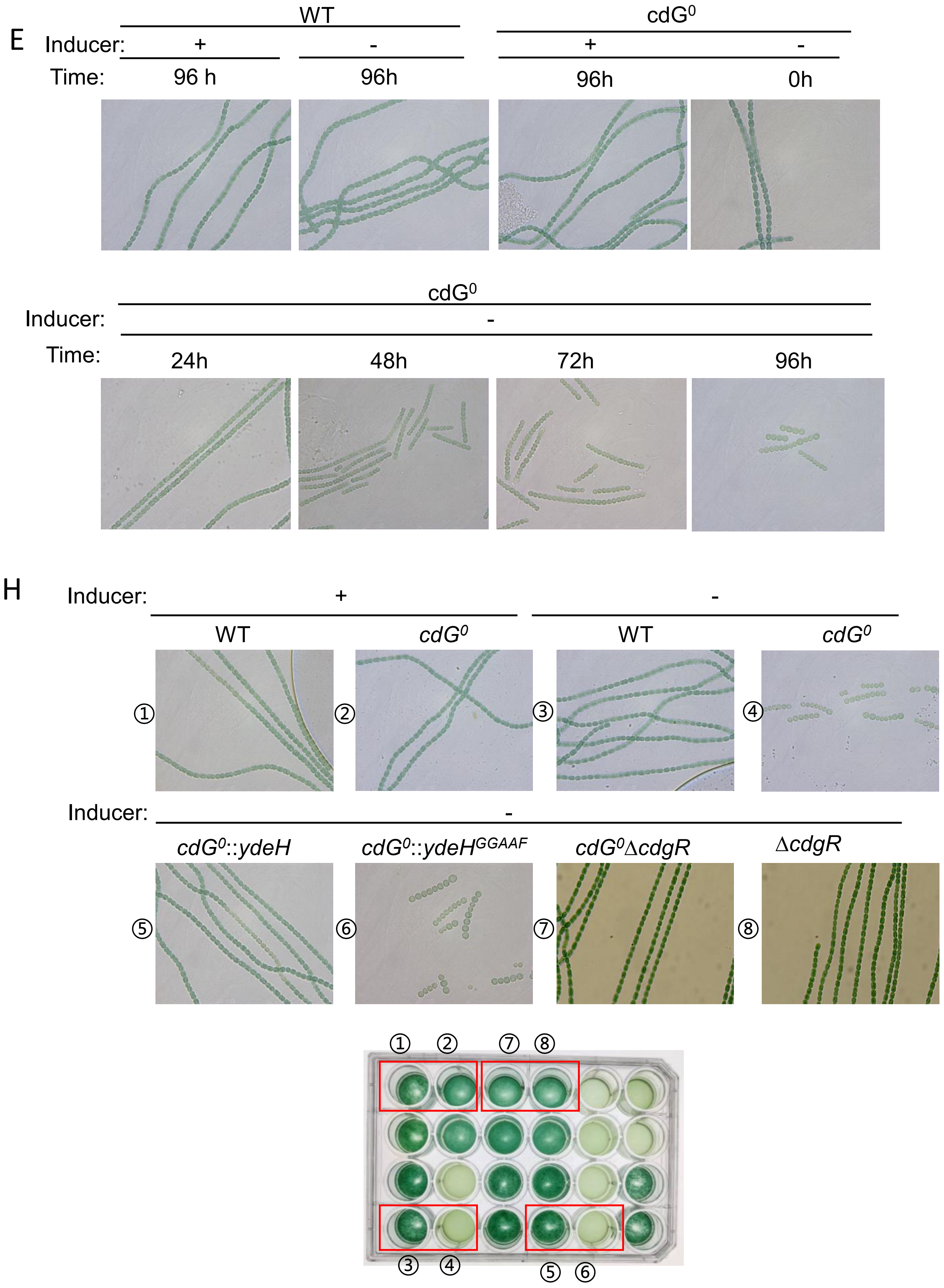
E
WT
cdG0
Inducer:
+
-
+
-
Time:
96 h
96h
96h
0h
cdG0
Inducer:
-
Time:
24h
48h
72h
96h
H
Inducer:
+
-
WT
cdG0
WT
cdG0
①
②
③
④
Inducer:
-
cdG0::ydeH
cdG0::ydeHGGAAF
cdG0ΔcdgR
ΔcdgR
⑤
⑥
⑦
⑧
① ② ⑦ ⑧
③ ④ ⑤ ⑥

Fig. 4

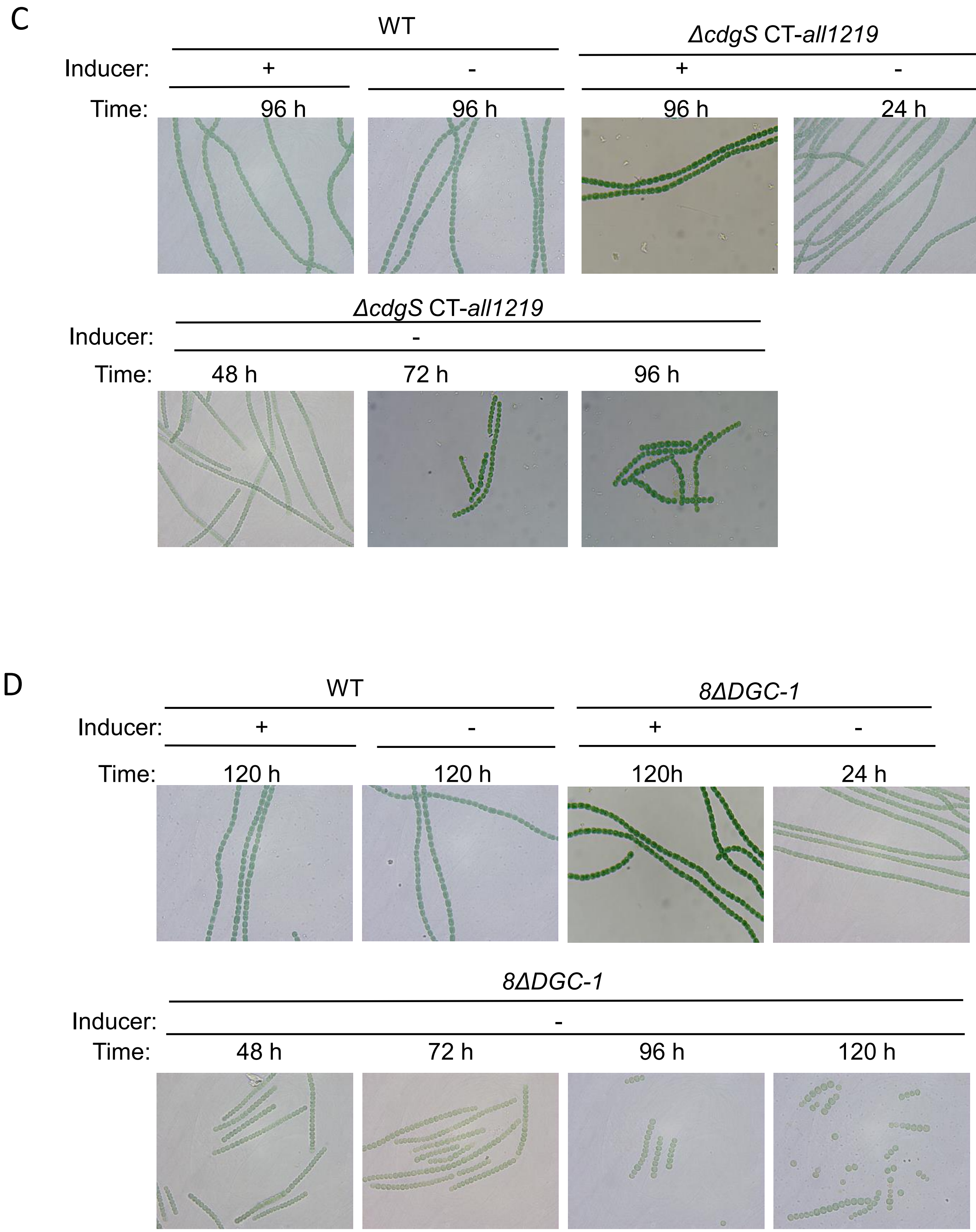
C
WT
ΔcdgS CT-all1219
Inducer:
+
-
+
-
Time:
96 h
96 h
96 h
24 h
ΔcdgS CT-all1219
Inducer:
-
Time:
48 h
72 h
96 h
D
WT
8ΔDGC-1
Inducer:
+
-
+
-
Time:
120 h
120 h
120h
24 h
8ΔDGC-1
Inducer:
-
Time:
48 h
72 h
96 h
120 h

Fig.4

E

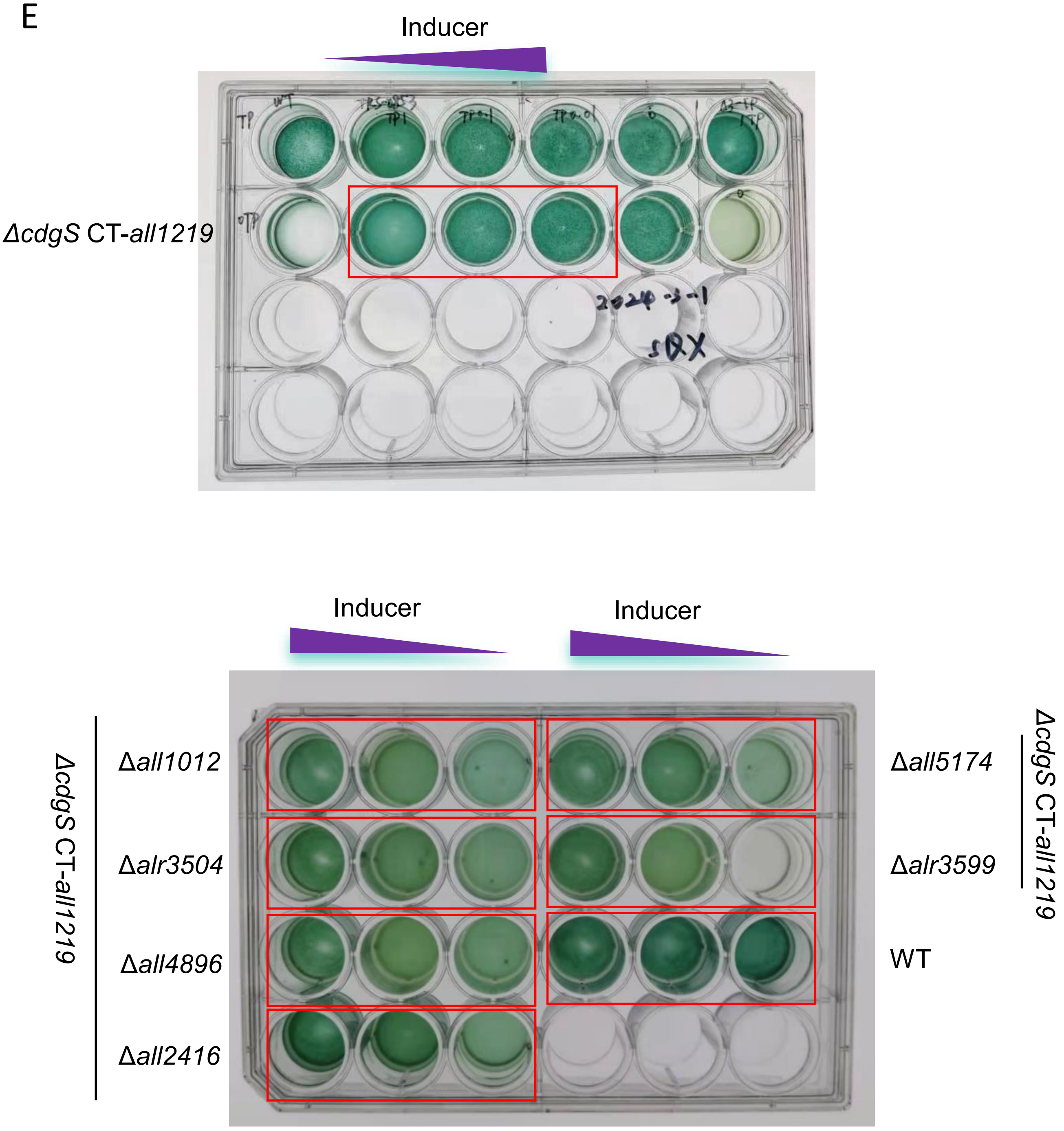
Inducer
ΔcdgS CT-all1219
Inducer
Inducer
ΔcdgS CT-all1219
Δall1012
Δalr3504
Δall4896
Δall2416
Δall5174
Δalr3599
WT
ΔcdgS CT-all1219

Fig. 5

D

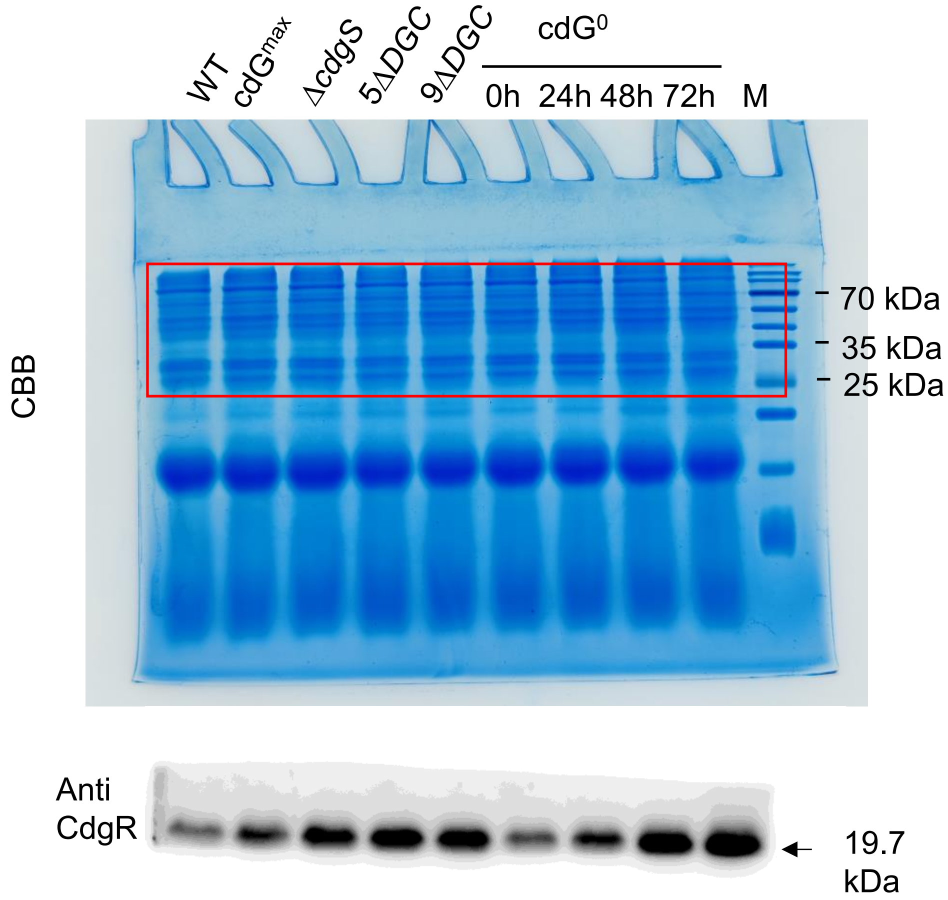

WT
cdG^max
ΔcdgS
5ΔDGC
9ΔDGC
cdG0
0h 24h 48h 72h
M
CBB
70 kDa
35 kDa
25 kDa
Anti CdgR
19.7 kDa

Fig. s1

B

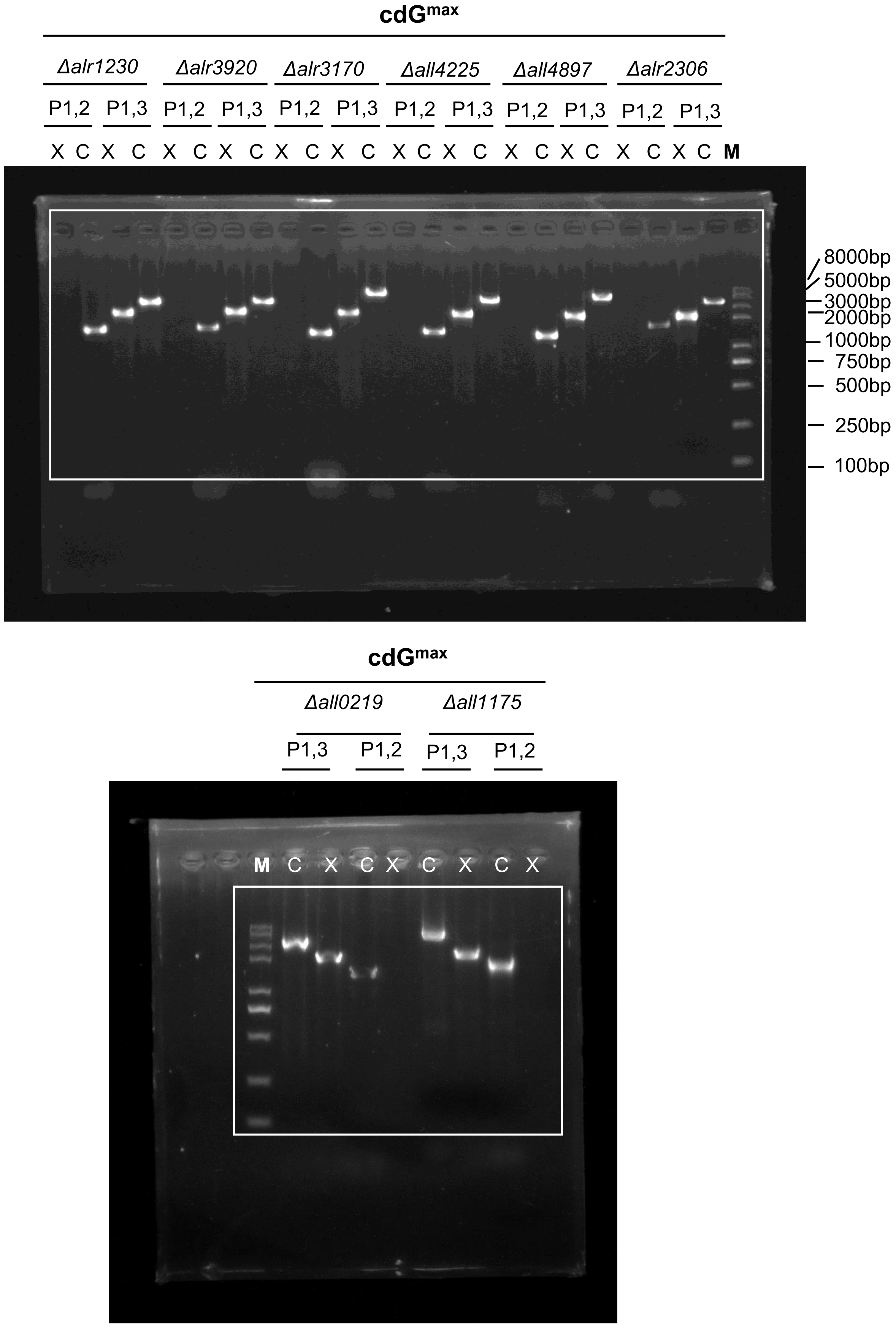

cdGmax
Δalr1230
Δalr3920
Δalr3170
Δall4225
Δall4897
Δalr2306
P1,2 P1,3 P1,2 P1,3 P1,2 P1,3 P1,2 P1,3 P1,2 P1,3 P1,2 P1,3
X C X C X C X C X C X C X C X C X C X C X C X C M
8000bp
5000bp
3000bp
2000bp
1000bp
750bp
500bp
250bp
100bp
cdGmax
Δall0219
Δall1175
P1,3 P1,2 P1,3 P1,2
M C X C X C X C X

Fig. s2

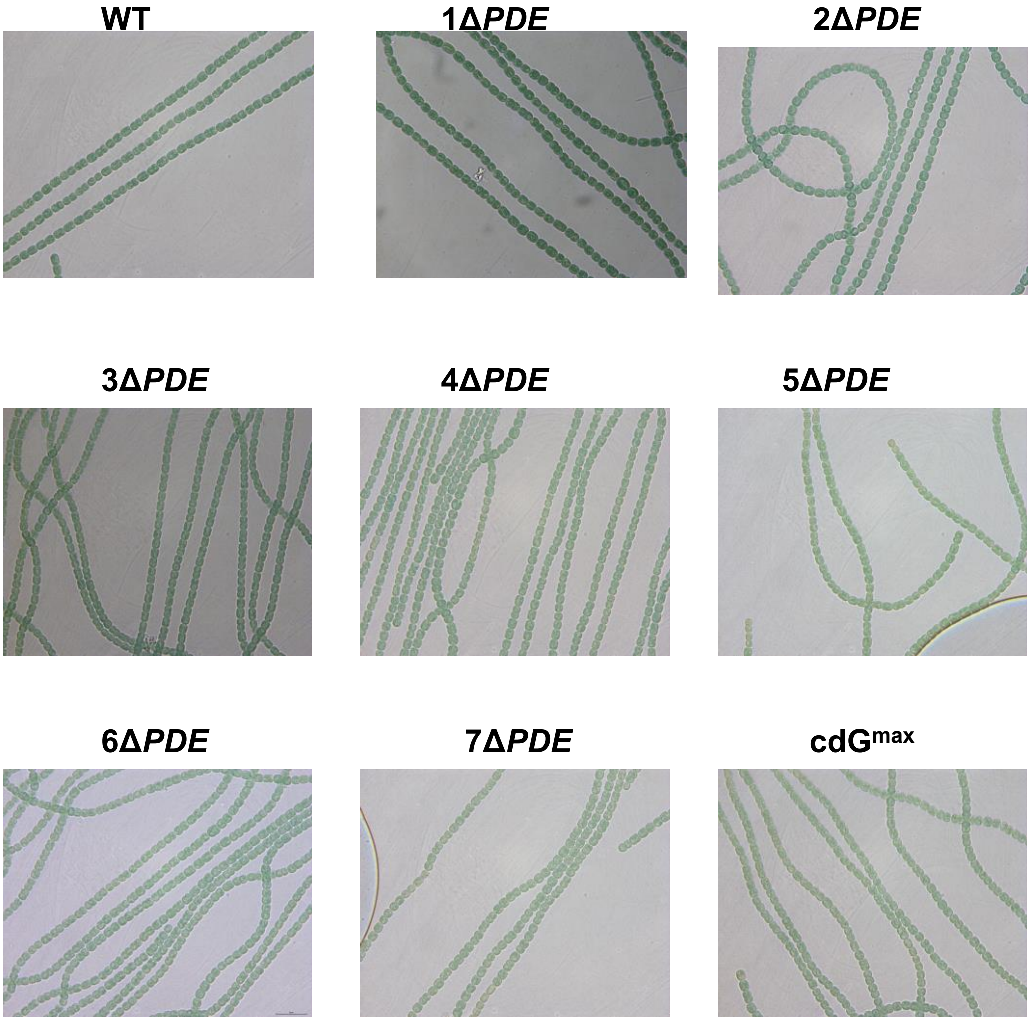

Fig. s4

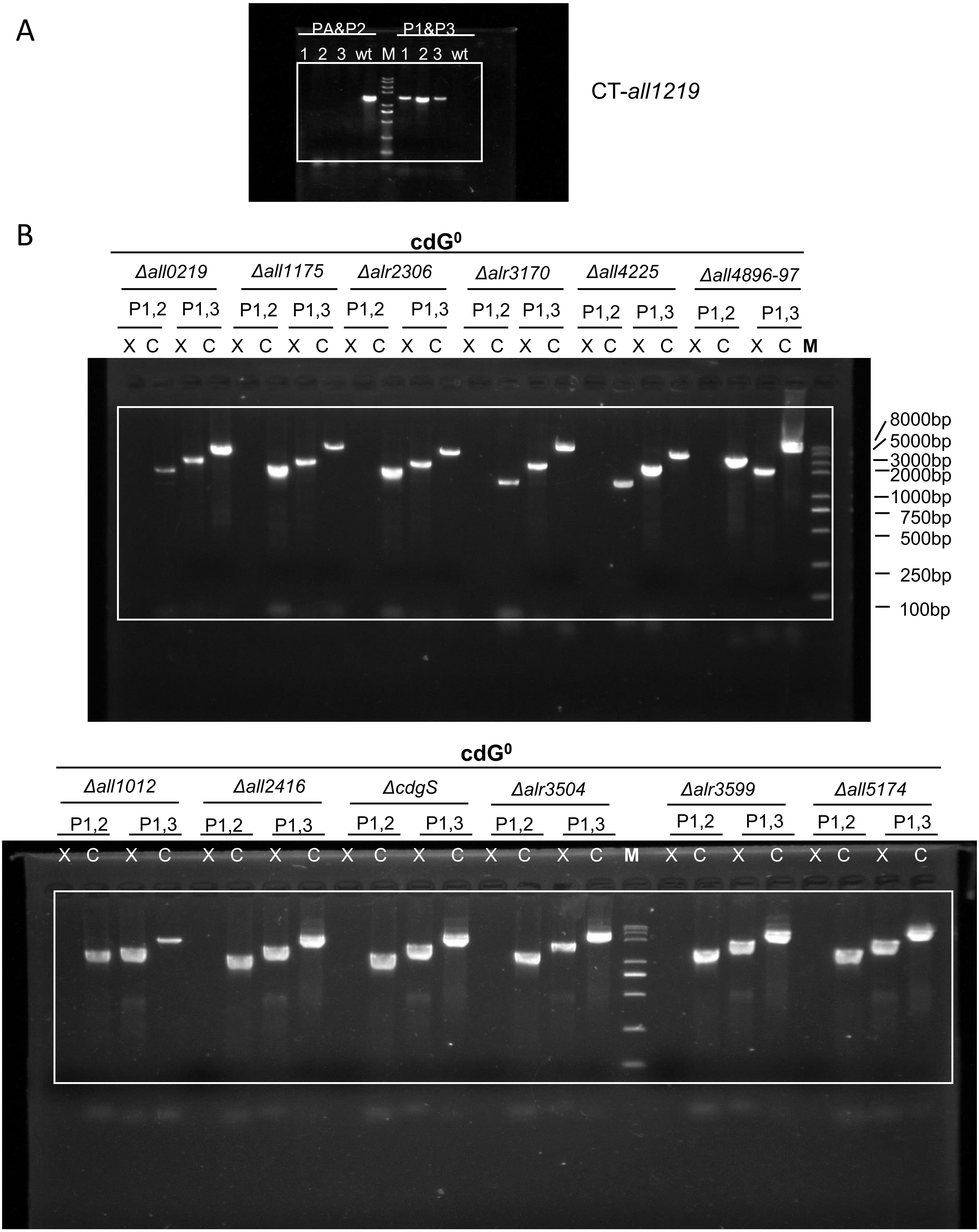
A
PA&P2
P1&P3
1 2 3 wt M 1 2 3 wt
CT-all1219
B
cdG0
Δall0219
Δall1175
Δalr2306
Δalr3170
Δall4225
Δall4896-97
P1,2 P1,3 P1,2 P1,3 P1,2 P1,3 P1,2 P1,3 P1,2 P1,3 P1,2 P1,3
X C X C X C X C X C X C X C X C X C X C X C X C M
8000bp
5000bp
3000bp
2000bp
1000bp
750bp
500bp
250bp
100bp
cdG0
Δall1012
Δall2416
ΔcdgS
Δalr3504
Δalr3599
Δall5174
P1,2 P1,3 P1,2 P1,3 P1,2 P1,3 P1,2 P1,3 P1,2 P1,3 P1,2 P1,3
X C X C X C X C X C X C X C X C M X C X C X C X C

Fig. s4

C

WT | *c*dG^0

Inducer + - + -

8 days

20 days

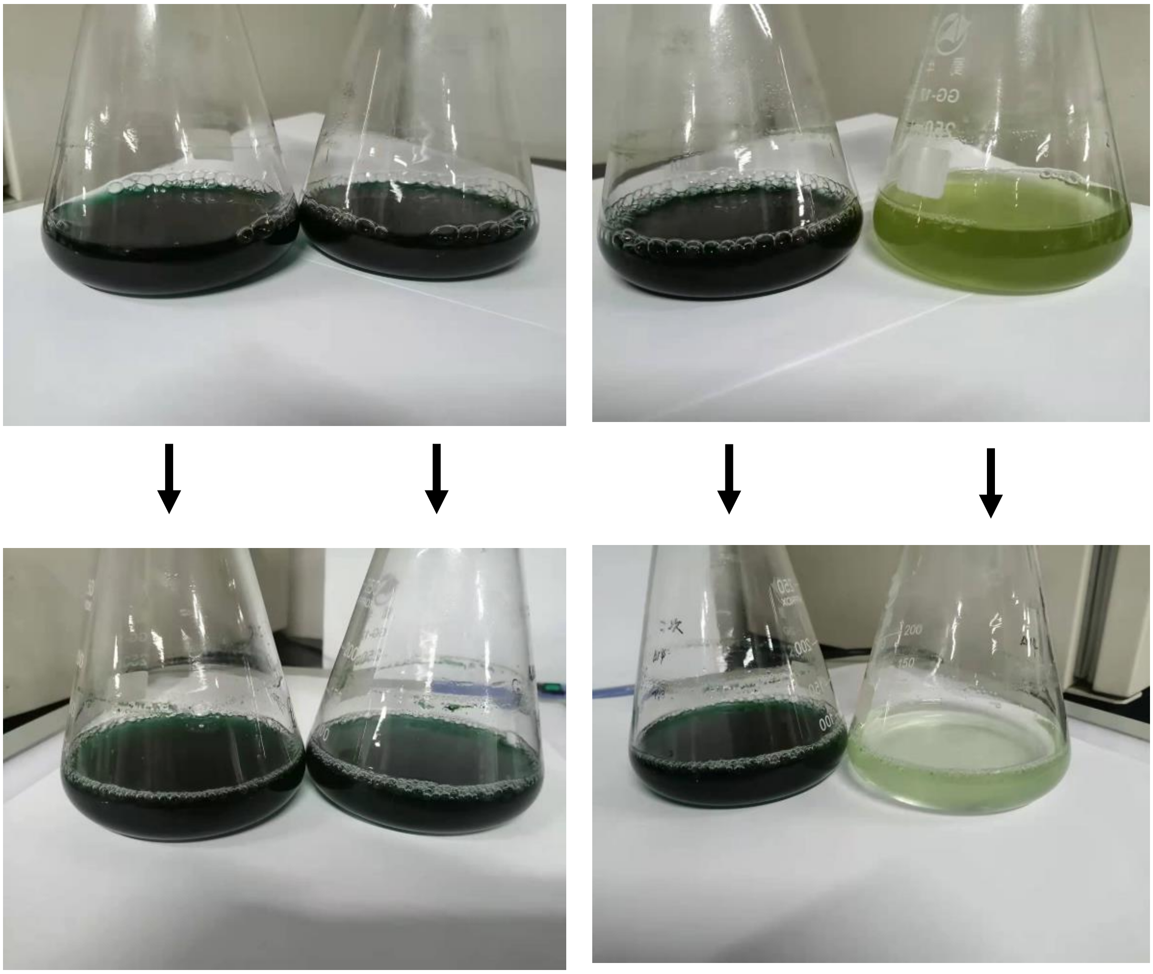

Fig. s5

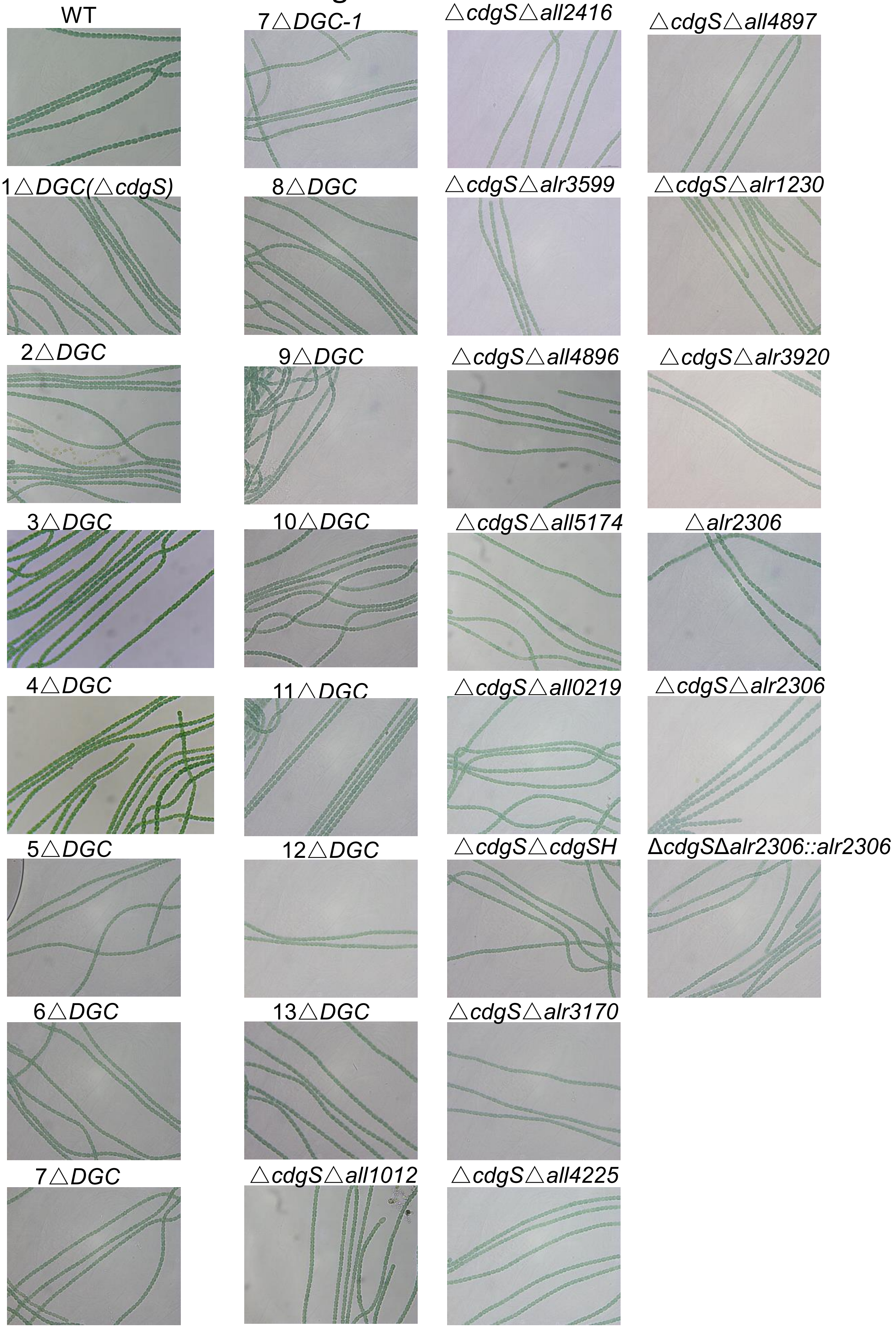
WT
1△DGC(△cdgS)
2△DGC
3△DGC
4△DGC
5△DGC
6△DGC
7△DGC
7△DGC-1
8△DGC
9△DGC
10△DGC
11△DGC
12△DGC
13△DGC
△cdgS△all1012
△cdgS△all2416
△cdgS△alr3599
△cdgS△all4896
△cdgS△all5174
△cdgS△all0219
△cdgS△cdgSH
△cdgS△alr3170
△cdgS△all4225
△cdgS△all4897
△cdgS△alr1230
△cdgS△alr3920
△alr2306
△cdgS△alr2306
ΔcdgSΔalr2306::alr2306

Fig. s6

E

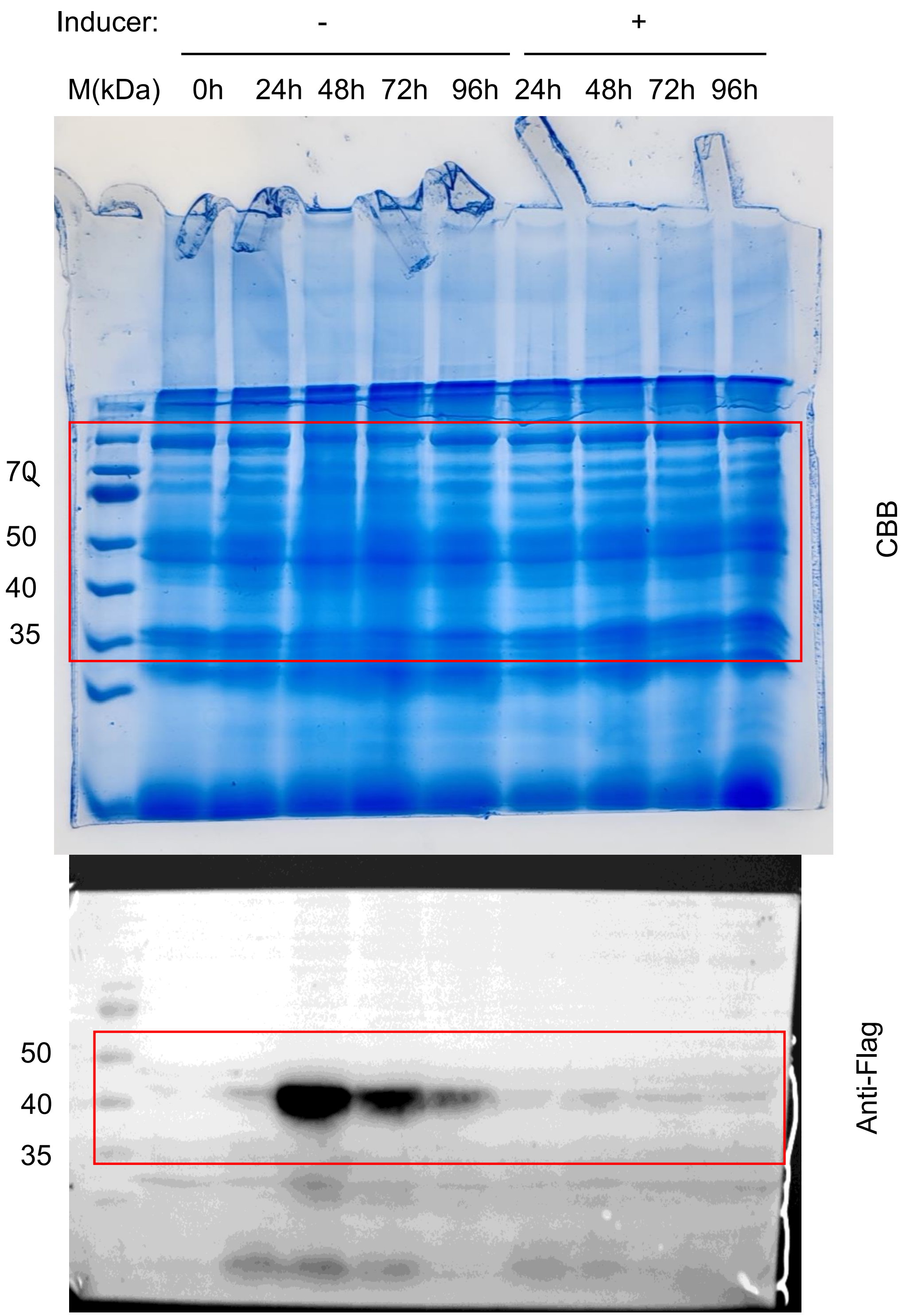

ΔcdgS CT-*all1219* (*alr3599*-Flag)

Fig. s7

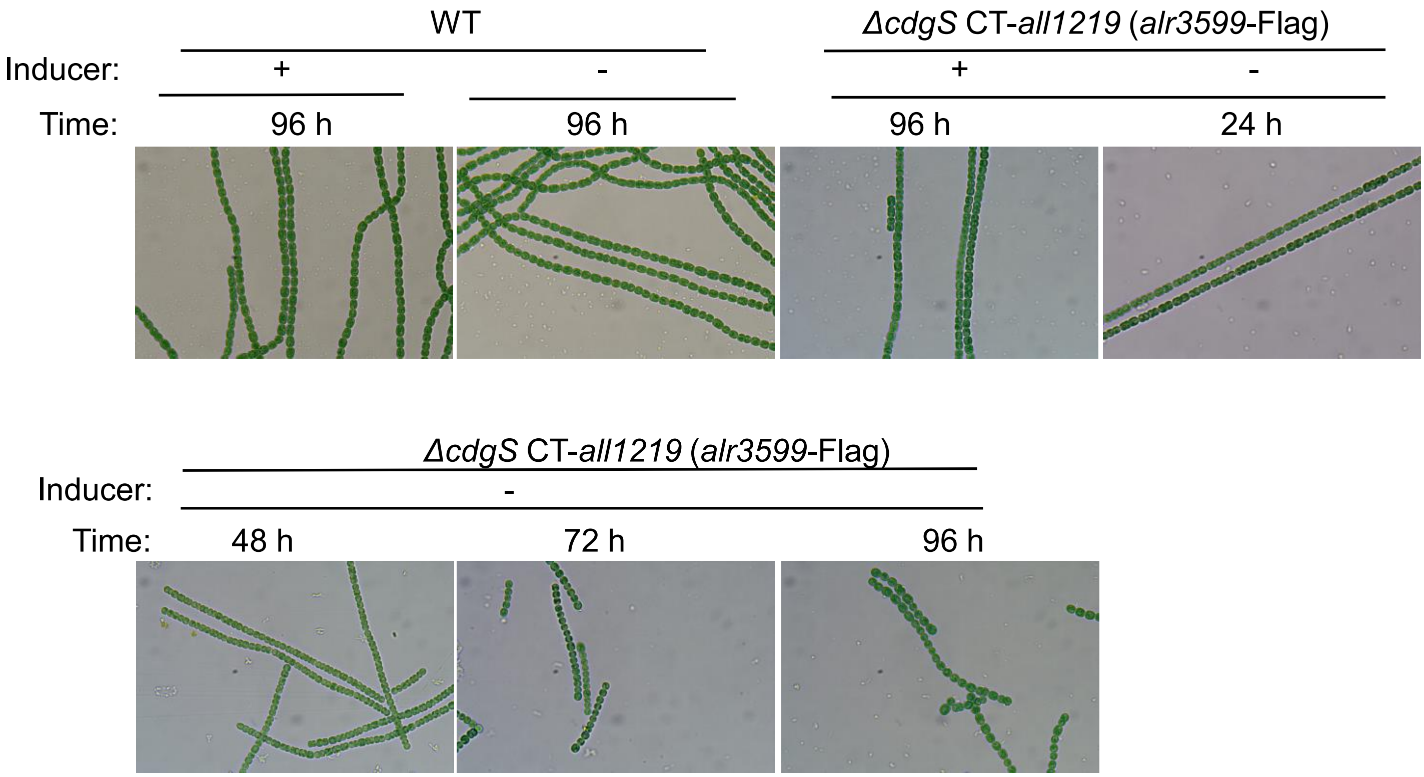

WT
ΔcdgS CT-all1219 (alr3599-Flag)
Inducer:
+
-
+
-
Time:
96 h
96 h
96 h
24 h
ΔcdgS CT-all1219 (alr3599-Flag)
Inducer:
-
Time:
48 h
72 h
96 h

Fig. s8

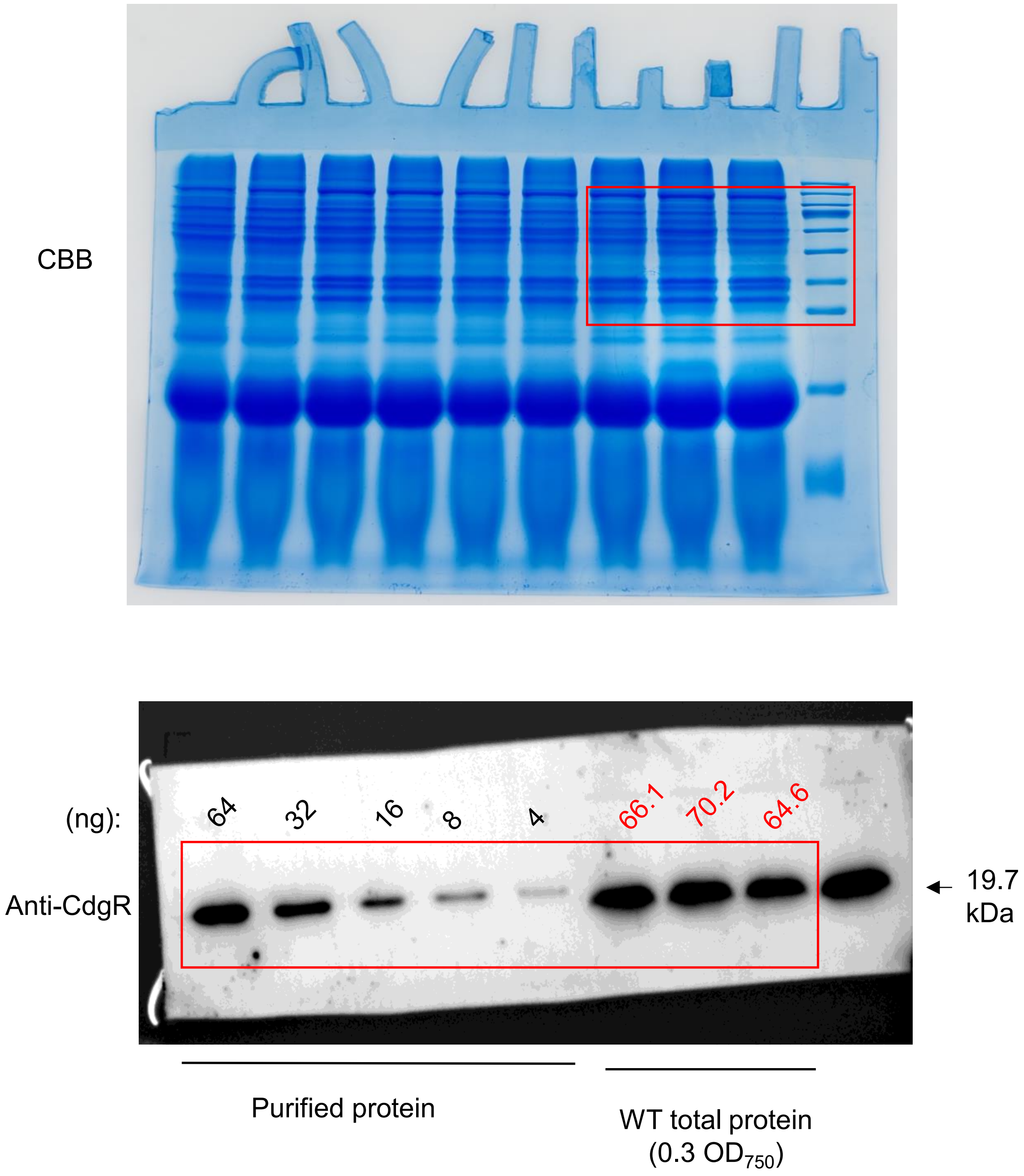
CBB
(ng):
64
32
16
8
4
66.1
70.2
64.6
Anti-CdgR
19.7 kDa
Purified protein
WT total protein
(0.3 OD750)

Fig. s9

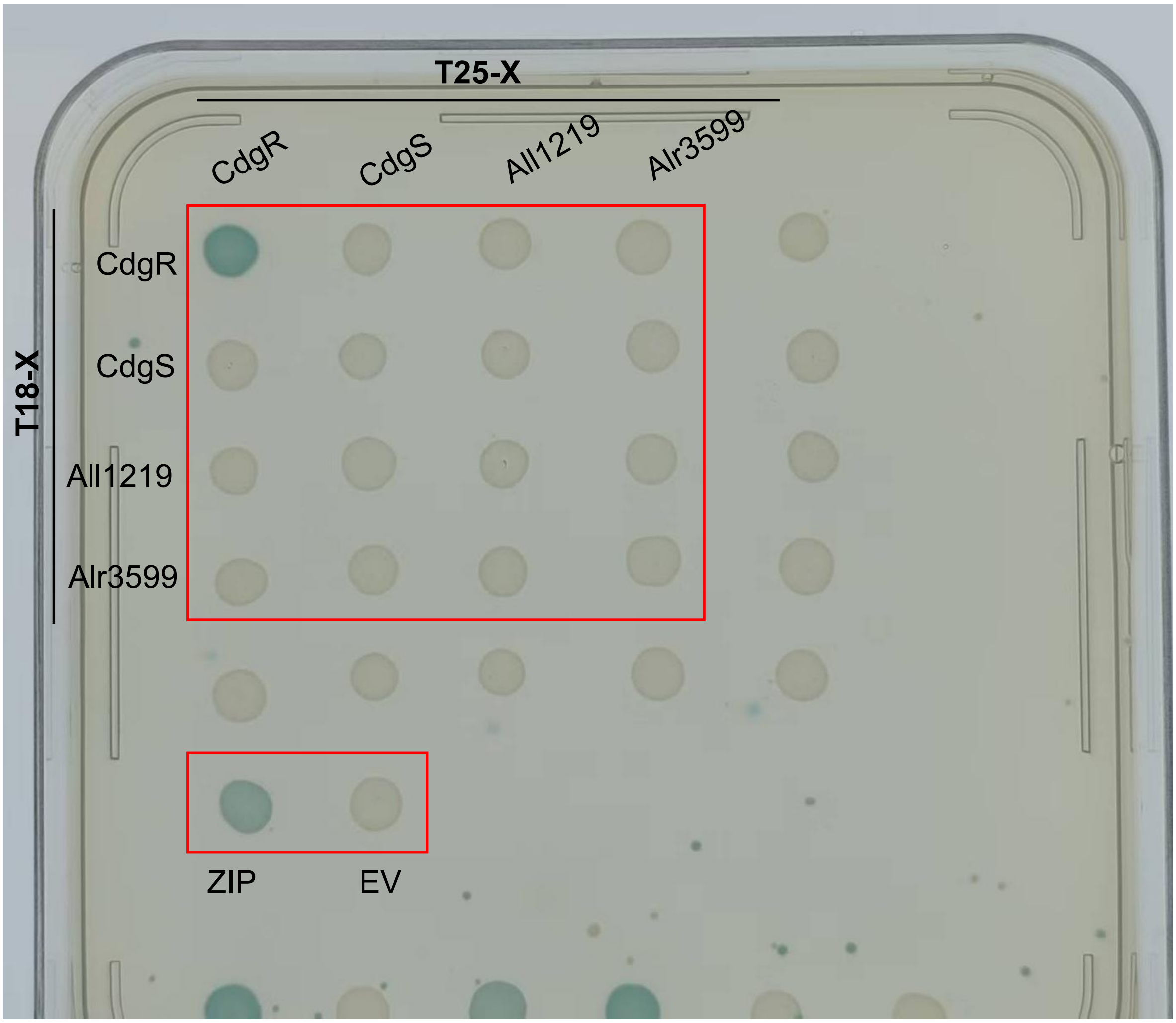

T25-X
CdgR
CdgS
All1219
Alr3599
T18-X
CdgR
CdgS
All1219
Alr3599
ZIP
EV

Fig. s10

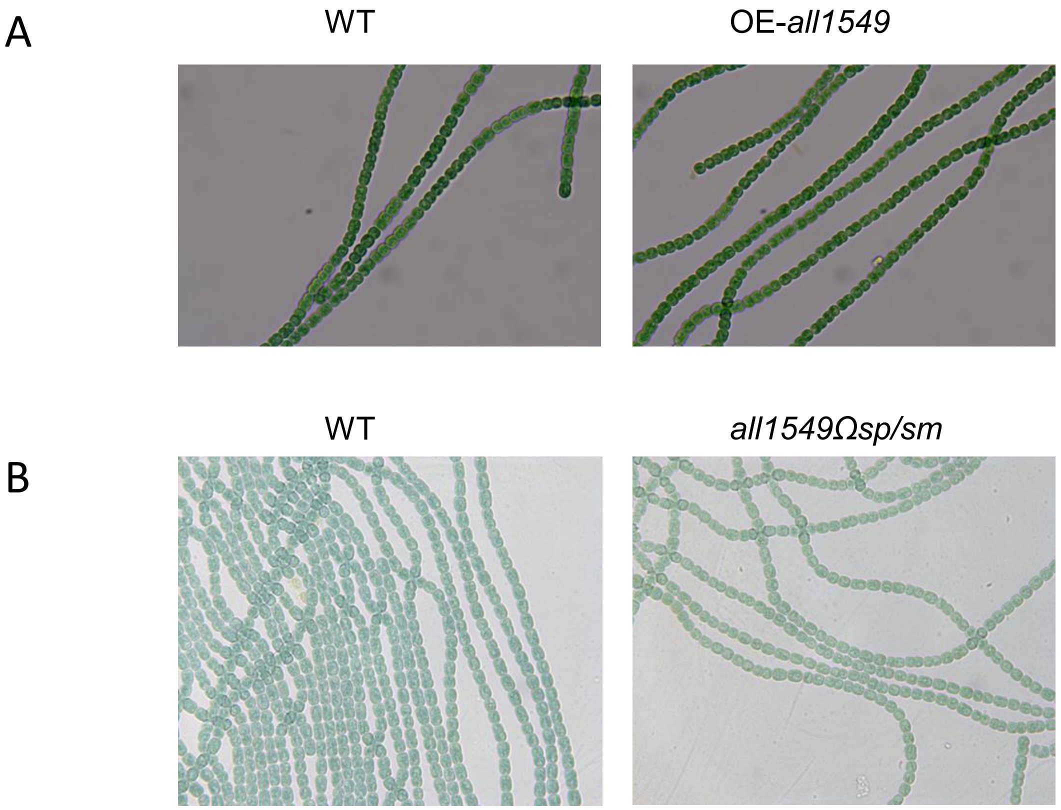

Supplement: S1 Raw Images — (PDF) [file pbio.3003750.s015.pdf]
