# Supplementary material for: A dual-threshold system relying on multiple c-di-GMP metabolic enzymes controls cell fate of a cyanobacterium
Source: PLoS Biol. 2026 Apr 8;24(4):e3003750. doi: 10.1371/journal.pbio.3003750 (PMC13075795; doi:10.1371/journal.pbio.3003750)
Supplement: Attachment — Submitted filename: point to point response.docx [file pbio.3003750.s018.docx]

Reviewer #1:

In this study, Sun et al probe the regulation of level of the bacterial second messenger c-di-GMP in the filamentous cyanobacterium Anabaena by constructing various kinds of mutants in 16 genes encoding enzymes responsible for c-di-GMP synthesis and/or c-di-GMP breakdown. C-di-GMP levels are measured in the mutants, and phenotypic effects are assessed by measuring growth rates and cell dimensions. There is also kinetic analysis of the enzymatic activity of some of the proteins. The experimental work all appears to have been done to a very high standard, and this is a technical tour de force - the construction, for example, of a mutant with simultaneous deletion of 14 genes is unprecedented in Anabaena, which is slow-growing, multicellular and polyploid. The main challenge is then trying to make a coherent story out of such a mountain of data. Here there is room for improvement. The authors' large collection of mutants enabled them to titrate c-di-GMP levels vs phenotype, and show the range of c-di-GMP levels over which c-di-GMP influences cell dimensions (Fig 4). They could further show a threshold level of c-di-GMP above which it no longer has any further effect on cell dimensions, and a second threshold below which further depletion of c-di-GMP is lethal. This is very interesting, and together with the data on the heterologous expression of a DGC (Fig 1 G,H) it strongly suggests that the multiple c-di-GMP metabolising enzymes in Anabaena exert their effects by acting as independent inputs into a global c-di-GMP pool, in contrast to the more usual picture of highly specific and localised c-di-GMP effects. For me that is the main message of the work. The authors discuss it (lines 569-584) but overall I think they need to be a lot clearer on what their study shows and what remains to be understood. Specific points:

Response to the above general comments:

Thank you for your positive evaluation of our work and for your valuable suggestions. We have revised the discussion section to clarify the key conclusions of this study and to point out what remains to be understood. See the discussion section in the revised manuscript.

1.	The authors make a big feature of their analogy with an electromechanical relay, which features in the title and takes up a whole figure (Fig. 5) and many lines of the discussion. It would be interesting to know what other reviewers made of it, but for me it didn't help at all - in fact I found it made the message harder to understand, as I had to try to understand a very complex and imperfect analogy in addition to trying to understand the system in Anabaena. I am not sure about lumping 13 enzymes together as a "basal power unit". One enzyme could do that job - isn't it more likely that all these enzymes are regulated differently to provide independent inputs into a global c-di-GMP pool? That would make this an interesting system for integrating information from multiple sensors. In any case, I would recommend that the authors find another way to present their model for the Anabaena system.

Response:

We apologize for any confusion caused. To develop a model that could comprehensively represent both the multi-enzyme metabolic system and the dual-threshold regulatory mechanism, we tried to put it in different models to help us and other readers better understand the whole story. At the end, the proposed model, which may not be perfect, still represents a good model for integrating different data into the system. However, we do agree that the complexity of this analogy may have obscured the core biological insights. In the revised manuscript, we have simplified and refocused the model description. Specifically, we changed the terminology, which should better help biologists to understand the model. For example, we changed "basal power unit" to “basal pool” of c-di-GMP, etc., which is more in line with biological languages. See more in lines 537-556 in the discussion section in the revised manuscript.

Regarding the classification of the 13 enzymes as a "basal functional unit," we would like to clarify that this interpretation specifically applies to the standard laboratory conditions examined in this study. We fully agree with the reviewer's point that under different environmental or signaling conditions, these enzymes are likely to be differentially regulated, thereby serving as distinct inputs influencing the global c-di-GMP pool, with potential changes in their functional categorization. We have emphasized this limitation in the revised discussion part, see lines 560-565.

We sincerely thank the reviewer for the insightful and constructive feedback regarding the electromechanical relay model.

2.	The discussion focuses on the phenotypes of mutants vs the wild type, but has nothing to say about regulation of cell size in the wild type. How plastic is cell size in wild type Anabaena and is there evidence for changes in global c-di-GMP levels in wild type Anabaena according to conditions? Is there any evidence for programmed cell death in Anabaena (as would be induced by very low c-di-GMP levels)? It is possible, as related species form necridia, for example to liberate hormogonia from the branched filament network in Fischerella/Mastigocladus species.

Response:

Thank you for raising these insightful and important questions. We agree that understanding the plasticity of cell size in wild-type Anabaena and the potential environmental regulation of c-di-GMP levels would greatly enhance the physiological relevance of our findings. However, we have not yet identified a direct environmental signal that triggers cell size changes via c-di-GMP modulation, and this remains an active area of our ongoing investigation. Your comments have been particularly enlightening. The extreme cell size reduction phenotype observed in our ultra-low c-di-GMP mutants (such as strains overexpressing PDE) aligns remarkably well with the reviewer’s hypothesis that extremely low c-di-GMP levels may induce PCD. This drastic reduction in cell size and condensation of cellular content may be a pathological state or initial phase leading to cellular collapse and death. Consequently, signals that trigger these processes may also be potential candidates for inducing cell size reduction. This perspective provides a valuable new direction for our future exploration of the environmental factors that regulate cell size in Anabaena. We have incorporated this discussion into the revised manuscript (see lines 557–586 in the Discussion section). Once again, we sincerely appreciate your thoughtful and constructive comments.

3.	Construction of mutants. Anabaena is polyploid and so it important to check for homozygosity of null mutants, whether they are generated by CRISPR or by any other method. Authors from the same group make that point in ref 46. Here, I assume that each null mutation was checked for full segregation, but, in the paper, nothing is said about that - information about how this was done must be provided.

Response:

Thanks for these constructive suggestions. We have now: (1) added a detailed description of the mutant construction and validation process in the Materials and Methods section (see lines 658-662 in the revised manuscript, and (2) included the PCR-based genotyping verification results confirming the complete segregation and homozygosity of the key *cdG^max^* and *cdG^0^* mutant strains as Supplementary Figures S1 and S4B (see Figure S1 and S4 in the revised manuscript).

4.	In their discussion the authors focus on cell dimensions, as that is what they look for, but it seems very likely that c-di-GMP controls other things too - I think that should be acknowledged in the discussion.

Response:

Thanks for this comment. We fully agree that the c-di-GMP pathway we have identified may also influence other cellular functions beyond cell size control directly and indirectly in *Anabaena*. In the revised discussion (see lines 572-577 in the revised manuscript), we have acknowledged this broader context.

5.	I found it impossible to keep track of the authors' arguments without constant reference to Table S1, which lists the genes involved and the mutants constructed. So that is essential information - it belongs in the main paper, not the supplementary. In fact it would be useful to add an extra column to Part A with comments on conclusions on regulation and function from this study.

Response:

Thanks for this comment. We realized that the lists of genes involved and the mutants constructed—originally presented in Figure S1—are essential for following the experimental logic. Accordingly, we have moved this information to the main text and, as recommended, added a column summarizing the conclusions on regulation and function derived from this study, see figure 1 in the revised manuscript.

6.	Lines 470-471: "c-di-GMP pool dynamics remain poorly characterized, particularly in cyanobacteria". That ignores a substantial literature in unicellular cyanobacterial species, which deserves more discussion here. See eg ref 4.

Response

Thanks for this comment. We agree that our original statement did not sufficiently acknowledge the existing research on c-di-GMP in unicellular cyanobacteria. Our intended point was to highlight the lack of a systemic understanding of how the entire c-di-GMP metabolizing enzyme system coordinately regulates the cellular c-di-GMP pool. To clarify this point, we have revised the sentence as follows (see lines 483-487 in the revised manuscript):

**Original:** “Most bacterial genomes encode numerous enzymes for c-di-GMP synthesis and degradation (16, 20, 23). However, the mechanisms governing c-di-GMP pool dynamics remain poorly characterized.”

**Modified:** “Most bacterial genomes encode numerous enzymes for c-di-GMP synthesis and degradation (16, 20, 23). It is also known that cyanobacteria modulate the c-di-GMP pool in a dynamic manner (4, 5, 16). However, a systemic understanding of how the entire enzyme system coordinately regulates c-di-GMP homeostasis remains lacking in cyanobacteria.”

7.	Line 101 - should be "homeostasis"?

Fixed.

8.	Line 263 - "maintaining" is confusing, as it suggests that Alr2306 is supplying c-di-GMP when in fact it seems to be acting mainly to break it down. "Controlling" would be better.

Response:

Thanks for this comment. We have changed "maintaining" to "controlling", seeing line? in the revised manuscript.

9.	Line 244, Fig. 2 legend. "p < 0.000" ??

Response

We sincerely apologize for the error in the legend of Fig. 2. We have changed "p < 0.000" to "p < 0.0001", seeing line? in the revised manuscript.

10.	Lines 566-568: "Employing threshold-dependent state transitions resolves the fundamental growth survival trade-off that challenges most living systems" (and a similar statement at the end of the abstract). I don't understand what that means. Is there even a growth-survival trade-off in this situation?

Response

Thanks for this question. To improve clarity and focus, we have deleted the sentences from the relevant section in the text and from the end of the abstract.

11.	Line 615. More details are needed on illumination, as light has been shown to have strong effects on c-di-GMP metabolism in cyanobacteria (eg ref 4).

Response

Thanks for this comment. We have added the details of the illumination, seeing line 635 in the revised manuscript.

12.	Line 668. Missing reference.

Added.

------------------------------------

Reviewer #2

The manuscript by Sun et. al. describes a comprehensive study of the role of c-di-GMP related genes in the filamentous cyanobacterium Anabaena (Nostoc) 7120. Using a comprehensive genetic approach they sequentially inactivate all of the genes responsible for c-di-GMP synthesis or degradation, demonstrating that a minimal threshold of c-di-GMP is required for cell viability, and that a second, higher threshold acts as a switch regulating cell size. The authors also identify key genes involved in maintaining c-di-GMP homeostasis that ensure levels do not drop below the critical threshold for viability. Overall, this is an impressive study in its thorough, comprehensive genetic approach to exploring the role of c-di-GMP in Anabaena 7120. The manuscript is generally well written, with a few minor editorial concerns listed below, and the results largely support the authors conclusions. The only major concern I have with the manuscript is that it fails to address how the loss of motility and biofilm formation in this organism due to laboratory domestication might obscure key roles for several of these genes. As written, the authors seem to imply that cell size and viability are the only two phenotypes regulated by c-di-GMP in this filamentous cyanobacterium. While this might be true for the commonly used lab strain, it may not reflect the entirety of the role of c-di-GMP in natural isolates of this strain and other closely related filamentous cyanobacteria. Other studies have demonstrated key roles for c-di-GMP in motility and biofilm formation for other filamentous cyanobacteria and it would be worth discussing all of this. On a related note, have the authors considered the possibility that the link between c-di-GMP and cell size regulation may be related to activation of dormant hormogonium development? Hormogonium development is known to be accompanied by a rapid reduction in cell size and shape, and while 7120 has lost the ability to differentiate hormogonia, it is possible that part of this pathway is activated by the decreased c-di-GMP levels, which could explain the biological relevance of cell size control via c-di-GMP.

Response to the above general comments:

Thanks for your positive evaluation of our work and for your valuable suggestions. Regarding the functions of c-di-GMP in *Anabaena*, we fully agree with your perspective. We recognize that our initial discussion did not address the fact that long-term laboratory cultivation has led to the loss of phenotypes such as motility and biofilm formation in this strain, thereby overlooking the potential involvement of c-di-GMP in regulating these functions. We have now revised the discussion section to address this point. It should be noted that whether c-di-GMP truly regulates these potential traits remains to be further investigated. The main challenge currently lies in the difficulty of obtaining reliable wild-type Anabaena strains for comparative analysis. Furthermore, the reviewer’s insightful suggestion regarding the possible link between c-di-GMP and cell size regulation through the activation of dormant hormogonium development pathways is highly inspiring. Although *Anabaena* has lost the ability to differentiate hormogonia, certain components of this pathway might still be regulated by c-di-GMP levels, thereby influencing cell size. This perspective provides a valuable new direction for our further exploration of environmental factors that regulate cell size in *Anabaena*. In response to the comments, we have incorporated relevant discussion in the revised manuscript, see lines 568-582. Once again, we sincerely thank the reviewer for the insightful comments.

Fig. 1D - are the cdG0 strains mislabeled, strain with inducer (green line) fails to grow, but strain without inducer (pink line) does grow? Opposite of what is reported in text and inconsistent with other data.

Response

We sincerely apologize for the mislabeling in the original Fig. 1D. The green line should be the strain without inducer, and the pink line should be the strain with inducer. We have now corrected this error in the revised manuscript, see Fig. 2D in the revised manuscript.

Line 186-187 - should this be figure 1D? Fig. 1C measures c-di-GMP levels, not growth.

Fixed.

Line 212 - insert "a" between "in" and "detectable"

Fixed.

Line 276-277 - As the authors note, it is surprising that removal of inducer does not lead to lack of cell growth in the DcdgSCT-all1219 strain, given that a cdgS-all1219 double mutant could not be generated. Could the authors comment on why this might be the case?

Response

Thanks for raising this important question. We speculate that this phenomenon may be related to the differences between solid and liquid culture conditions. During gene knockout screening on solid media, the growth conditions are typically more stringent. The cdgS-all1219 double knockout may cause the strain to fail to form colonies on plates due to excessive environmental stress, where cells might perish before they can activate the emergency system to restore c-di-GMP levels. In contrast, under liquid culture or conditional gene suppression conditions, the environment is relatively milder, providing physiologically vulnerable strains with a time window to sustain basic survival. This enables them to initiate emergency response mechanisms and gradually resume growth. This difference suggests that whether the loss of gene function leads to phenotypic lethality may highly depend on the environmental pressure and adaptation time faced by the cells.

Line 373 - insert "during" between "homeostasis" and "standard"

Fixed.

Line 427 - would "phenotypic consequences" be more appropriate than "genetic consequences" here?

Response

Thanks for this comment. We have changed the "genetic consequences" to" phenotypic consequences", see line 438.in the revised manuscript.

442 - Is apo-form the appropriate term here? Apo-form typically refers to the inactive form of an enzyme, but here it seems that it is the form that does not have c-di-GMP bound that is the active form, leading to growth arrest, and that c-di-GMP is an inhibitor of cdgR.

Response

Thanks for this comment. While 'apo-form' is structurally accurate for the ligand-free state, we agree that it can be misleading in this study, as the c-di-GMP-free form is the active form. To prevent any misconception, we have changed the “apo-form” to “the c-di-GMP free form” in the revised manuscript for explicit expression, see lines 42, 453, 462, 471, 479.

Line 453 - why does reduced c-di-GMP lead to increased levels of CdgR? Is the mechanism known? If not, could the authors speculate on what why this occurs here or in the discussion?

Response

Thanks for this comment. The mechanism is indeed not yet known. To acknowledge this open question, we have added a sentence “The mechanism underlying this CdgR accumulation upon c-di-GMP depletion remains to be determined, but may involve c-di-GMP-mediated repression of cdgR expression or inhibition of CdgR protein degradation.” in the results section, noting that the accumulation could be due to transcriptional or post-translational regulation, and that the exact mechanism remains a subject for future study. See lines 465-468 in the revised manuscript.

Line 577-591 - Given that laboratory isolates of this organism are not known to display motility or biofilm formation, it is perhaps not surprising that perturbing c-di-GMP levels would not influence these phenotypes. However, this is likely due to other mutations which have resulted in the loss of these phenotypes in PCC 7120.

Response

Thanks for this comment. We have addressed this point in detail in our general response. We fully agree that the long-term laboratory cultivation of the Anabaena PCC 7120 strain has likely led to other genomic mutations resulting in the loss of phenotypes such as motility and biofilm formation. Therefore, the absence of observed c-di-GMP regulation over these phenotypes in our study likely reflects the genetic background constraints of this lab strain rather than an inherent lack of function within the c-di-GMP signaling pathway. This is a crucial point that will help readers interpret our findings more accurately. We have added a discussion of this limitation to the revised manuscript, see lines 568-582.
